# Protective effect of tin chloride on rhabdomyolysis-induced acute kidney injury in rats

**DOI:** 10.1371/journal.pone.0265512

**Published:** 2022-03-16

**Authors:** Shinkichi Ohtani, Hiroko Shimizu, Masakazu Yamaoka, Toru Takahashi, Emiko Omori, Hiroshi Morimatsu

**Affiliations:** 1 Department of Anesthesiology and Resuscitology, Okayama University Graduate School of Medicine, Dentistry and Pharmaceutical Sciences, Okayama, Japan; 2 Department of Anesthesiology and Resuscitology, Okayama University Medical School, Okayama, Japan; 3 Department of Anesthesiology, Japanese Red Cross Society Himeji Hospital, Hyogo, Japan; 4 Department of Faculty of Health and Welfare Science, Okayama Prefectural University, Okayama, Japan; Zagazig University, EGYPT

## Abstract

The heme component of myoglobin plays a crucial role in the pathogenesis of rhabdomyolysis-associated acute kidney injury (RM-AKI). Heme oxiganenase-1 (HO-1) is the rate-limiting enzyme of heme catabolism, and its metabolites, iron, biliverdin, and carbon monoxide, have antioxidant properties. Tin chloride (SnCl_2_) is a kidney specific HO-1 inducer. In this study, we examined whether the induction of HO-1 in the kidney by SnCl_₂_ pretreatment ameliorates RM-AKI in rats and if the effect is due to the degradation of excess renal free heme. We developed an RM-AKI rat (male Sprague-Dawley rats) model by injecting glycerol (Gly) in the hind limbs. RM-AKI rats were pretreated with saline or SnCl_₂_ or additional SnMP (tin mesoporphyrin, a specific HO inhibitor) followed by Gly treatment. Serum blood urea nitrogen (BUN) and creatinine (Crea) were measured as indicators of renal function. Renal free heme level was assessed based on the levels of δ-aminolevulinate synthase (ALAS1), a heme biosynthetic enzyme, and nuclear BTB and CNC homology 1 (Bach1), an inhibitory transcription factor of HO-1. Elevated free heme levels lead to decreases in ALAS1 and nuclear Bach1. After 24 h of Gly injection, serum BUN and Crea levels in saline-pretreated rats were significantly higher than those in untreated control rats. In contrast, SnCl_₂_-pretreated rats showed no significant increase in the indices. However, additional treatment of SnMP abolished the beneficial effect of SnCl_₂_. Renal ALAS1 mRNA levels and renal nuclear Bach1 protein levels in the saline pretreated rats were significantly lower than those in control rats 3 h after Gly injection. In contrast, the levels in SnCl₂-pretreated rats were not altered. The findings indicate that SnCl_2_ pretreatment confers protection against RM-AKI by virtue of HO-1 induction in the renal system, at least in part through excess free heme degradation.

## Introduction

Rhabdomyolysis is a life-threatening condition characterized by extensive muscle damage leading to the expression of myoglobin. This leads to severe oxidative damage, ultimately manifesting as acute kidney injury [1]. In rhabdomyolysis-associated acute kidney injury (RM-AKI), the heme component of myoglobin can stimulate lipid peroxidation due to redox cycling of the heme group from ferrous to ferric and then to ferryl oxidation states, causing renal injury [1]. However, few strategies for protecting renal cells from heme-mediated oxidative damage other than symptomatic treatments, such as fluid infusion therapy and renal replacement therapy, have been developed, and progress in protection against RM-AKI is eagerly awaited.

Heme oxygenase-1 (HO-1), which is the rate-limiting enzyme in the catabolism of heme, is activated by heme and other stimuli [2,3]. Numerous studies have shown that HO-1 induction has a protective effect on cells against oxidative injuries, including RM-AKI [4–8]. Previous studies have reported that tin chloride (SnCl_2_) is a kidney-specific inducer of HO-1 [9,10]. We also previously demonstrated that SnCl_2_ pretreatment significantly ameliorated ischemic kidney injury in rats through the kidney-specific induction of HO-1 [10]. However, the protective effect of SnCl_2_ has not been elucidated in other types of AKI, including RM-AKI.

In a previous study, we developed a RM-AKI rat model by injecting glycerol in the hind limbs. We proposed that there was a marked rise in intracellular heme concentration in the renal system of RM-AKI rats based on dynamic changes in three substances, including HO-1, δ-aminolevulinate synthase (ALAS1)—a key enzyme involved in heme metabolism that is usually downregulated by heme—and the nuclear Broad-complex, Tramtrack and Bric-a-brac (BTB), and cap’n’collar (CNC) homology proteins 1 (Bach1), a heme responsive transcription factor, which is exported from the nucleus and binds to intracellular heme. In RM-AKI rats, under excess free heme, renal mRNA levels of ALAS1 are downregulated and renal nuclear bach1 protein levels decreases due to binding to intracellular heme, which lead to endogenous induction of HO-1 as a defense mechanism [11].

In the abovementioned context, we designed this study with two objectives. First, we aimed to investigate whether pharmacological induction of HO-1 expression by SnCl_2_ prevents RM-AKI in rats. Second, we aimed to determine whether the effect of SnCl_2_ pretreatment on ameliorating RM-AKI is mediated through heme metabolism. We hypothesized that pretreatment with SnCl_2_ would ameliorate RM-AKI by inducing HO-1 through heme metabolism.

## Materials and methods

### Animals

The animal experiments in the present study received approval from the Animal Use and Care Committee of Okayama University Medical School on July 30 (OKU-2014411), on October 30, 2017 (OKU-2017459), and on August 25, 2020 (OKU-2020473). The study adhered to the ARRIVE (Animal Research: Reporting of in vivo Experiments) guidelines for animal care and handling. Male Sprague-Dawley rats weighing 200–220 g were purchased from Clea Japan, Inc. (Tokyo, Japan). The rats were kept in a temperature-controlled room maintained at 25°C and under a 12 h light/dark photoperiod. Furthermore, rats were allowed free access to water and chow until the start of the experiments. Overall, 97 rats were used in the present study: (control group [n = 4], saline+Gly group [n = 4–8 at each of the five different time points, total = 25], SnCl_2_+Gly group [n = 4 at each of the five different time points, total = 20], SnCl_2_+SnMP+Gly group [n = 3–6 at each of the five different time points, total = 18], SnCl_2_ group [n = 3 at each of the five different time points, total = 15], and saline group [n = 3 at each of the five different time points, total = 15]). Sample size were decided according to previous study [11].

### Experimental design

**Fig 1:**
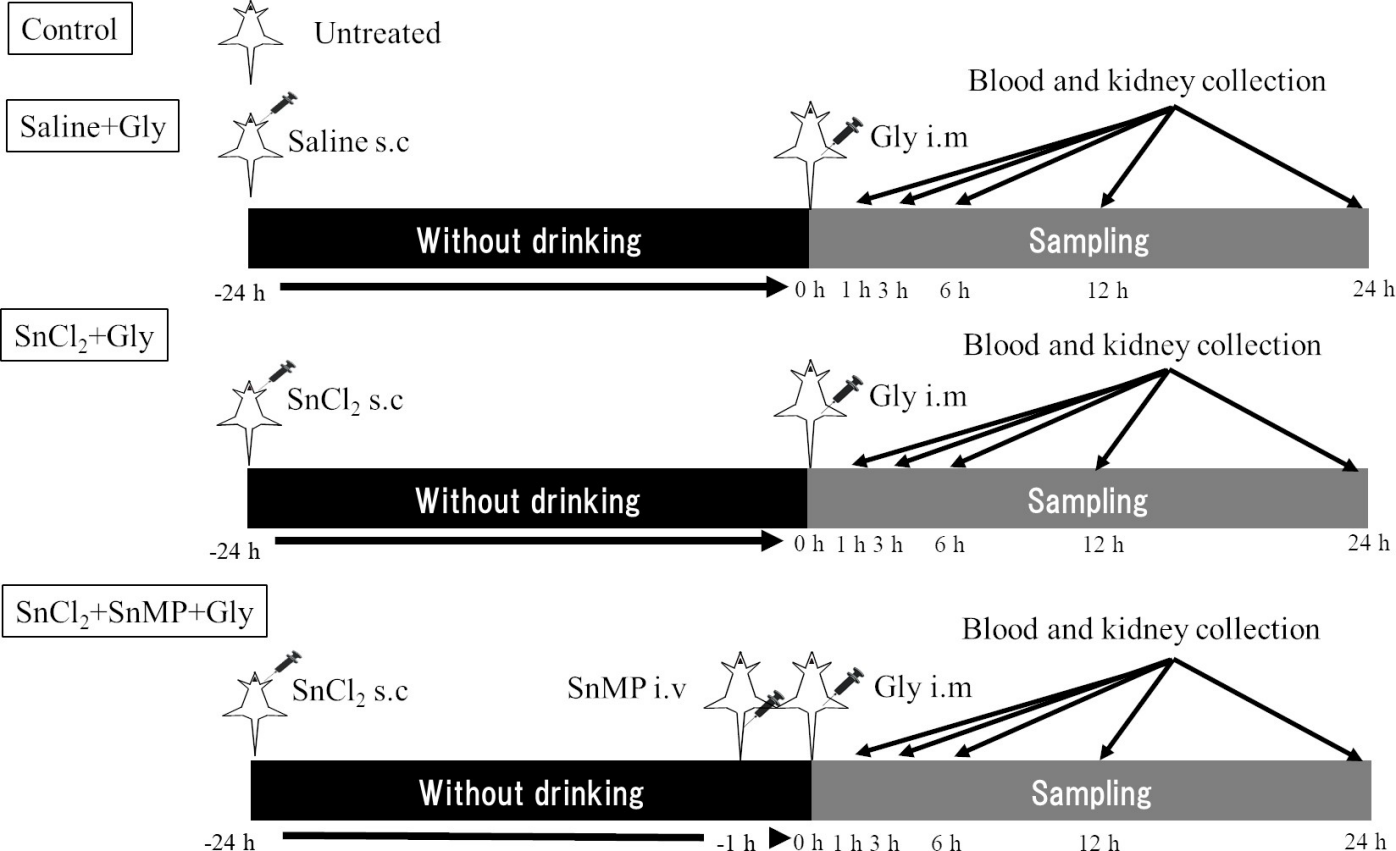
Experimental protocol and grouping. Rats were randomly divided into four groups: Control, saline+Gly, SnCl_2_+Gly, and SnCl_2_+SnMP+Gly. Rats in the control group were untreated. Rats in the other three group were treated with saline or SnCl_2_ (s.c.) and then deprived of water for 24 h. Rats in the SnCl_2_+SnMP+Gly group received an additional treatment of SnMP (i.v.) 1 h before Gly injection. 24 h after saline or SnCl_2_ treatment, rats were treated with Gly (i.m.); afterward, at five time points (1, 3, 6, 12, and 24 h) and their blood and kidneys collected.

This experimental protocol was prepared before the study. The rats were denied access to water for 24 h before being randomly divided into the following four groups (n = 3–8 in each group): control (untreated), saline+Gly, SnCl_2_+Gly, and SnCl_2_+SnMP (tin mesoporous, a specific HO inhibitor)+Gly. Rats in the SnCl_2_+Gly group received a subcutaneous injection (s.c.) with SnCl_2_ (10 mg/100 g body weight, dissolved in saline at a concentration of 100 mg/mL, the optimal dose for HO-1 induction in rats [10]), followed by an intramuscular injection (i.m.) of 50% glycerol (Gly) (10 mL/kg; Ishizu Seiyaku, Ltd, Osaka, Japan), the optimal dose for developing RM-AKI rats [11] in both hind limbs after 24 h. The rats belonging to the saline+Gly group were administered with the same amount of saline, followed by a Gly injection. In the SnCl_2_+SnMP+Gly group, the rats additionally received an intravenous injection (i.v.) of SnMP (1 μmol/kg, dissolved in 0.01 M sodium phosphate buffer, the optimal and non-harmful dose for inhibiting HO activity in rats [10]) 1 h before injecting Gly in the SnCl_2_+Gly group. After Gly injection, rats in all groups were provided access to drinking water and chow. Blood and kidneys were collected after Gly injection at each time point (1, 3, 6, 12, and 24 h) by opening the abdominal cavity under inhalation anesthesia with isoflurane in O_2_ (2% for induction, 1–1.5% for maintenance) and the individuals were euthanized by phlebotomy while maintaining the anesthesia. Blood was collected in heparinized centrifuge tubes through a catheter inserted into the abdominal aorta to evaluate biochemical values, and the kidneys were perfused with heparinized saline and then collected. The kidney specimens were quickly frozen using liquid nitrogen and kept at a temperature of −80°C for subsequent use in RNA or protein preparation. For histological analysis, kidney tissues were fixed in 10% neutral-buffered formalin and embedded in paraffin. This experimental protocol and grouping were graphically illustrated (Fig 1). In another set of experiments, to investigate whether HO-1 induction by SnCl_2_ was associated with free heme and to determine whether SnCl_2_ adversely affects biochemical properties, SnCl_2_ (n = 3) or saline (n = 3) were individually injected in intact animals. After injection, blood and kidney were collected at multiple time points (1, 3, 6, 12, and 24 h) using the same method as described above.

All rats were assigned a number in the order in which they were purchased and kept in cages of the same size and in the same location. The experiments were conducted in the same place in a fixed order each time. Group allocation and pretreatment of drugs were conducted by H Shimizu. Gly treatment, sampling, and data analysis were conducted by S Ohtani. Group allocation information was shared with S Ohtani after sampling. The inclusion criteria were rats that were alive and healthy, and the exclusion criteria were rats that had died unexpectedly or were extremely weak or suffering as described following. During the entire course of the experiment, including the 24-hour fasting period, if it was determined that the animal was in intolerable pain, for example, assessed based on ≥20% loss of body weight or decreased mobility, we defined as humane endpoints and the experiment was immediately stopped and the animal was euthanized with an overdose (200 mg/kg) of sodium pentobarbital (i.v.). Nevertheless, no rats fell into the category. Two rats unexpectedly died. One died 6 h after saline treatment, and another 3 h after SnCl_2_ treatment. All efforts were made to minimize animal suffering.

### RNA isolation and Northern blot analysis

Total RNA was isolated from the kidney tissues using TRI Reagent® (Molecular Research Center, Inc. Cincinnati, USA) according to the guidelines provided by the manufacturer. Northern blot analysis was carried out as previously reported [12]. Briefly, electrophoresis on a 1.2% (w/v) agarose gel with 6.5% (v/v) formaldehyde was used to separate total RNA (20 μg). After blotting on a Bio-Rad Zeta-Probe GT Blotting Membrane (Bio-Rad Laboratories, Richmond, CA, USA), the RNA was hybridized with [α-32P] dCTP (PerkinElmer, Inc., Japan)-labeled complementary DNA (cDNA) probe against HO-1 (provided by Dr. S Shibahara, Sendai University, Sendai, Japan) and ALAS1 (provided by Dr. M Yamamoto, Tohoku University, Sendai, Japan), followed by washing under appropriate conditions. Each blotted membrane was exposed to a sheet of Fuji Medical X-ray film (Fujifilm Co., Tokyo, Japan) with an intensifying screen at −80°C. Signals corresponding to the target mRNA on the film and 18S ribosomal RNA bands visualized on the gel were estimated using an image scanner (ChemiDoc XRS Plus Image Processing System, Bio-Rad, USA) and image analysis software (Image Lab™ version 5.0; Bio-Rad Laboratories). The relative amounts of hybridized radioisotope-identified cDNAs were normalized to 18S ribosomal RNA levels to correct for sample loading differences.

### Preparation of microsomal and nuclear-cytosolic fractionation

The kidney specimens were homogenized using Potter grinder in 0.05 M Tris-HCl (pH 7.8) with 0.25 M sucrose and centrifuged at 90,000 *g* for 10 min at 4°C. Then the supernatant was centrifuged at 105,000 *g* for 60 min at 4°C to separate the microsomal fraction. The pellet was resuspended in 20 mM Tris-HCl (pH 7.4) with 0.15 M KCl and used to measure HO activity. Nuclear and cytoplasmic proteins were extracted using NE-PER Nuclear and Cytoplasmic Extraction Reagents (Pierce, Rockford, IL, USA) based on the manufacturer’s protocol. Cytoplasmic proteins from kidney tissues were extracted by adding kidney homogenates in ice-cold cytoplasmic extraction reagent 1 (CER1) with a protease inhibitor (cOmplete; Roche Diagnostics GmbH, Mannheim, Germany) using a Dounce grinder, and then adding CER2 to the homogenate. After this, the mixture was centrifuged at 16,000 *g* for 5 min at 4°C to obtain the cytoplasmic fraction from the supernatant. Next, the nuclear proteins were extracted from the pellet with ice-cold nuclear extraction reagent (NER) containing a protease inhibitor, and the supernatant was centrifuged at 4°C for 10 min. Assessment of protein concentration was carried out using the BCA assay (Pierce, Rockford, IL, USA).

### Measurement of HO activity

Kidneys were homogenized in 0.05 M Tris-HCl (pH 7.8) with 0.25 M sucrose. Microsomal fractions were extracted as described above, and HO activity was evaluated spectrophotometrically, as previously reported [13,14]. As a source of biliverdin reductase, the cytosolic fraction from the livers of untreated adult rats was prepared. HO activity was calculated as picomoles of bilirubin formed per milligram of protein per 60 min.

### Western blotting

Western blot analysis was carried out according to previously reported protocols [15]. In brief, proteins (20 μg) from each sample were loaded on a 7.5% or 12.5% (w/v) sodium dodecyl sulfate-polyacrylamide gel. After electrophoresis, the proteins were placed on Amersham™ Hybond™ P 0.45 polyvinylidene fluoride membranes (GE Healthcare Japan Co., Tokyo, Japan) and blocked with 4% (w/v) BLOCK ACE^®^ (DS Pharma Biomedical Co., Ltd., Osaka, Japan) solution for 1 h at room temperature. The membranes were incubated overnight with the following primary antibodies diluted in Tris-buffered saline with Tween 20 (TBST) at 4°C: rabbit anti-HO-1 polyclonal antibody (1:1000 dilution, ADI-SPA-895-F, Enzo Life Science, Farmingdale, NY, USA), rabbit anti-Bach1 polyclonal antibody (1:1000 dilution, 14018-1-AP, Proteintech, Tokyo, Japan), mouse anti-β-actin monoclonal antibody (1:5000 dilution, sc-47778, Santa Cruz, Dallas, TX, USA), and rabbit anti-GAPDH antibody (1:5000 dilution, sc-25778, Santa Cruz). After washing with TBST, the membranes were incubated with secondary antibodies (mouse anti-rabbit IgG-HRP 1;20000 dilution, sc-2357, Santa Cruz for HO-1; goat anti-mouse IgG-HRP, 1:20000 dilution, sc-2005, Santa Cruz for β-actin; goat anti-rabbit IgG H&L [HRP], 1:20000 dilution, ab6721, Abcam, Tokyo, Japan, for Bach1, and GAPDH) for 30 min at room temperature. Antigen-antibody complexes were stained with Clarity Western ECL Substrate (Bio-Rad) and visualization was performed using an image scanner (ChemiDoc XRS Plus Imaging System, Bio-Rad). The signals were estimated using Image Lab Version 5.0 (Bio-Rad).

### Biochemical assays

The levels of blood urea nitrogen (BUN) and creatinine (Cr) and the activities of creatine phosphokinase (CPK) and alanine aminotransferase (ALT) in the serum were measured. Centrifugation at 1600 *g* lasting about 10 min at room temperature was used to separate the serum from the whole blood, and the parameters described above were analyzed using an automated biochemical analyzer (Fuji DRI-CHEM 7000i; Fujifilm Co., Tokyo, Japan).

### Histological analysis

Kidneys—prepared for histological analysis as described above—were sectioned to a thickness of 4–6 μm. After deparaffinization and dehydration, hematoxylin and eosin (HE) stain was administered to the sections and viewed using a light microscopy. Tubular injuries were assessed by four observers in a blinded manner. Tubular injury—such as tubular epithelial cell swelling, vacuolar degeneration, necrosis, and desquamation—was scored and graded based on the proportion of injured tubules in the following manner: 0, areas of injured tubules < 5%; 1, areas of injured tubules 5–25%; 2, areas of injured tubules 26–50%; 3, areas of injured tubules 51–75%; and 4, areas of injured tubules > 75%, as recommended in a previous report [16]. The evaluation was performed on 10 randomly selected areas per kidney and four kidneys per group. The results are shown as the average of the tubular injury scores for each group.

### Statistical analysis

Data are reported as mean ± standard deviation (SD). Statistical analyses were carried out using Student’s *t*-test to compare means between two groups, Analysis of Variance followed by Tukey-Kramer multiple comparisons to compare means among three or more groups, and Dunnett’s multiple comparison test to comparing means between the control group and other groups, using JMP Pro 14^®^software (SAS Institute Inc., Cary, NC, USA). Differences were considered significant at p < 0.05.

## Results

### Effect of SnCl_2_ pretreatment on the expression of HO-1 in the kidney of RM-AKI

**Fig 2:**
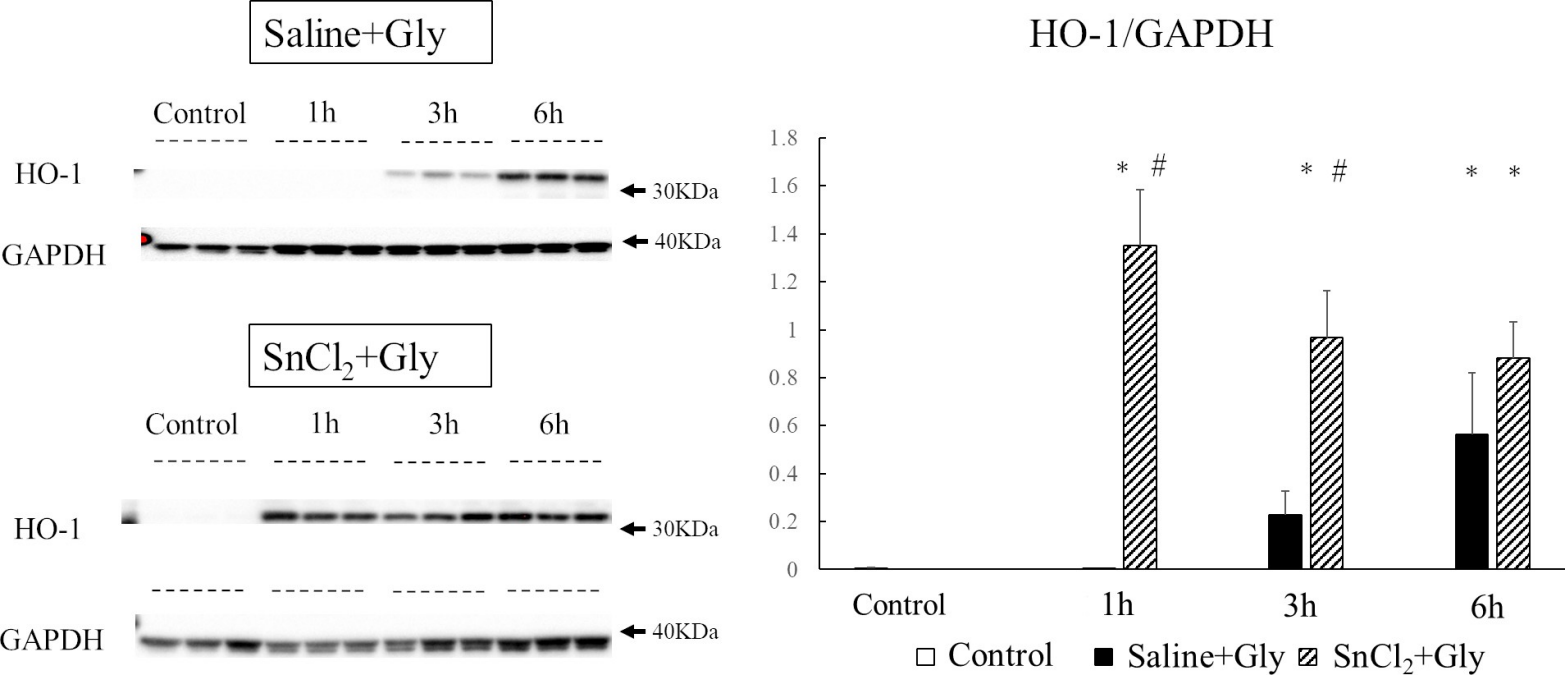
Changes in renal HO-1 protein expression after Gly treatment. Rats pretreated with saline or SnCl_2_ (s.c.) were sacrificed 1, 3, and 6 h after Gly treatment (10 mL/kg of 50% glycerol, i.m.), and renal tissues were collected for western blot analysis. Control, untreated control rats; saline+Gly, rats pretreated with saline before Gly injection; SnCl_2_+Gly, rats pretreated with SnCl_₂_ before Gly injection. The densitometric ratio between HO-1 and GAPDH was calculated. Data are expressed as the mean ± SD. *p < 0.05 vs control group. #p < 0.05 vs saline+Gly group.

**Fig 3:**
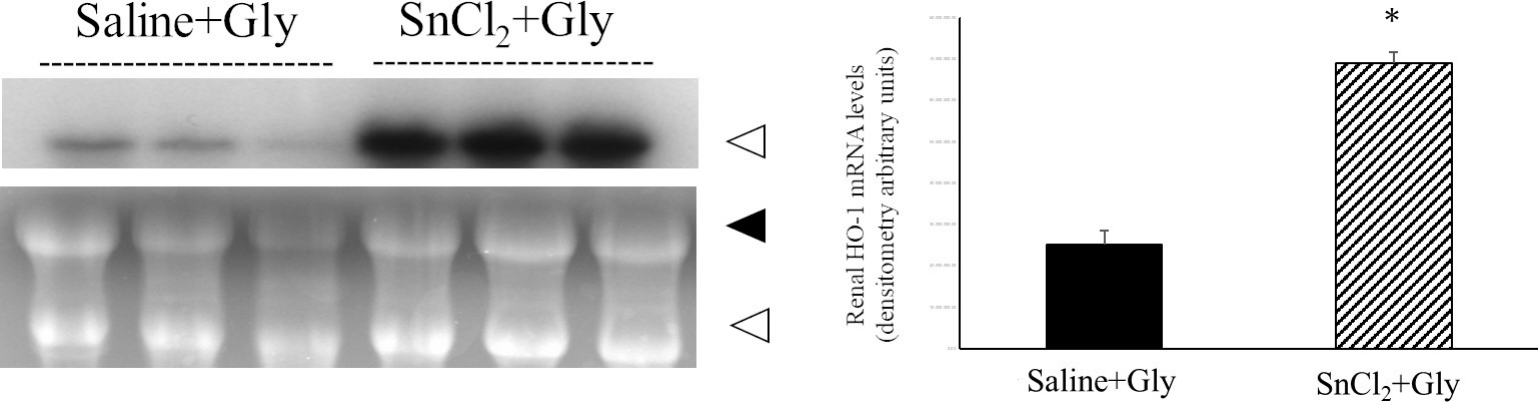
Expression of renal HO-1 mRNA 6 h after Gly treatment. Rats pretreated with saline or SnCl_2_ (s.c.) were sacrificed 6 h after Gly treatment (10 mL/kg of 50% glycerol, i.m.) and renal tissues were collected for northern blot analysis. Control; untreated control rats, saline+Gly; saline-treated before Gly-treated rats, SnCl_2_+Gly; SnCl_2_-treated before Gly-treated rats. Ethidium bromide staining of the same RNA is shown as a loading control. Closed arrowhead, 28S ribosomal RNA; open arrowhead, 18S ribosomal RNA. HO-1 mRNA expression levels are expressed as arbitrary densitometric units. Data are expressed as the mean ± SD. *p < 0.05 vs saline+Gly group.

First, we examined the effect of SnCl_2_ pretreatment on the renal protein expression of HO-1, the rate-limiting enzyme in heme catabolism, at 1, 3, and 6 h after Gly treatment. The protein levels of HO-1 in the saline-treated group were barely detectable at 1 h after treatment. Their levels started to increase at 3 h and then further increased at 6 h. In contrast, in SnCl_2_-treated animals, HO-1 protein levels were markedly upregulated, even at 1 h after Gly treatment, and these high levels were maintained at 6 h after the treatment (Fig 2). Next, to confirm that the enhanced HO-1 protein expression in SnCl_2_-treated animals is due to *de novo* synthesis of HO-1 in the kidney, we measured the gene expression of renal HO-1 in SnCl_2_-treated animals and compared it to that of saline-treated animals at 6 h after Gly treatment, the highest expression time point after Gly treatment [11]. Northern blot analysis revealed that renal HO-1 mRNA levels in the SnCl_2_-treated group were significantly higher than those in the saline-treated group (p < 0.0001) (Fig 3). These results indicate that SnCl_2_ pretreatment incited rapid and enhanced renal HO-1 expression compared to saline-pretreated animals in the early phase after Gly treatment.

### Effect of SnCl_2_ pretreatment on kidney injury in RM-AKI

**Fig 4:**
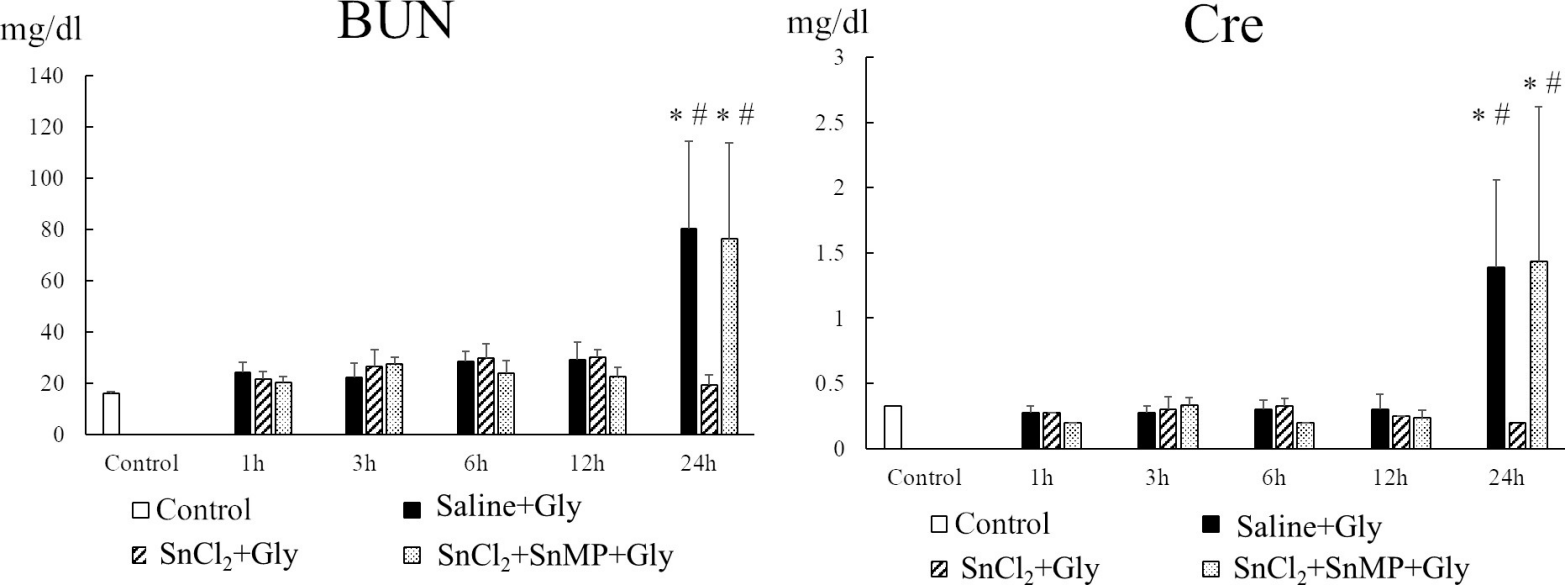
Effect of SnCl_2_ pretreatment on changes in serum BUN and Cre levels after glycerol treatment. Rats pretreated with saline (s.c.) or SnCl_2_ (s.c.) or SnCl_₂_ and SnMP (i.v.) 24 h before Gly treatment (10 mL/kg of 50% glycerol, i.m.) were sacrificed at 1, 3, 6, 12, and 24 h after Gly injection. Serum BUN and Cre levels were measured at each time after treatment. Control, untreated control rats; saline+Gly, saline-treated before Gly-treated rats; SnCl_2_+Gly, SnCl_2_-treated before Gly-treated rats; SnCl_2_+SnMP+Gly, SnCl_2_ and SnMP-treated before Gly-treated rats. Data are expressed as the mean ± SD (n = 3–7). * p < 0.05 vs control group; # p < 0.05 vs SnCl_2_+Gly group. BUN, blood urea nitrogen; Cre, creatinine.

Next, we examined whether SnCl_2_ pretreatment could ameliorate RM-AKI induced by glycerol injection via HO-1 induction in rats. The changes in the levels of serum BUN and creatinine with time were determined following the administration of glycerol in all three groups of rats: pretreatment with saline (saline+Gly), SnCl_2_ (SnCl_2_+Gly), and SnCl_2_ with SnMP, a specific competitive inhibitor of HO activity (SnCl_2_+SnMP+Gly). At 12 h after the administration of glycerol, both indices in each group were similar to those in the untreated control animals. Moreover, when the three groups were compared at each time point, the values of these indices in the three animal groups did not show statistical difference until 12 h after glycerol treatment. However, at 24 h after the glycerol treatment, there was a significant increase in serum BUN and creatinine levels in the saline+Gly group compared to those in the control group (BUN, 80.35 ± 34.25 mg/dl vs 15.9 ± 0.84 mg/dl, p < 0.0001; creatinine, 1.38 ± 0.67 mg/dl vs 0.32 ± 0.095, p = 0.025). In contrast, the SnCl_2_+Gly group exhibited significantly lower serum BUN and creatinine levels than the saline+Gly group (BUN, 19.47 ± 3.62 mg/dl vs 80.35 ± 34.25 mg/dl, p < 0.0001; creatinine, 0.2 ± 0 mg/dl vs 1.38 ± 0.67 mg/dl, p = 0.006). However, additional treatment with SnMP diminished the beneficial effects of SnCl_2_ (Fig 4).

**Fig 5:**
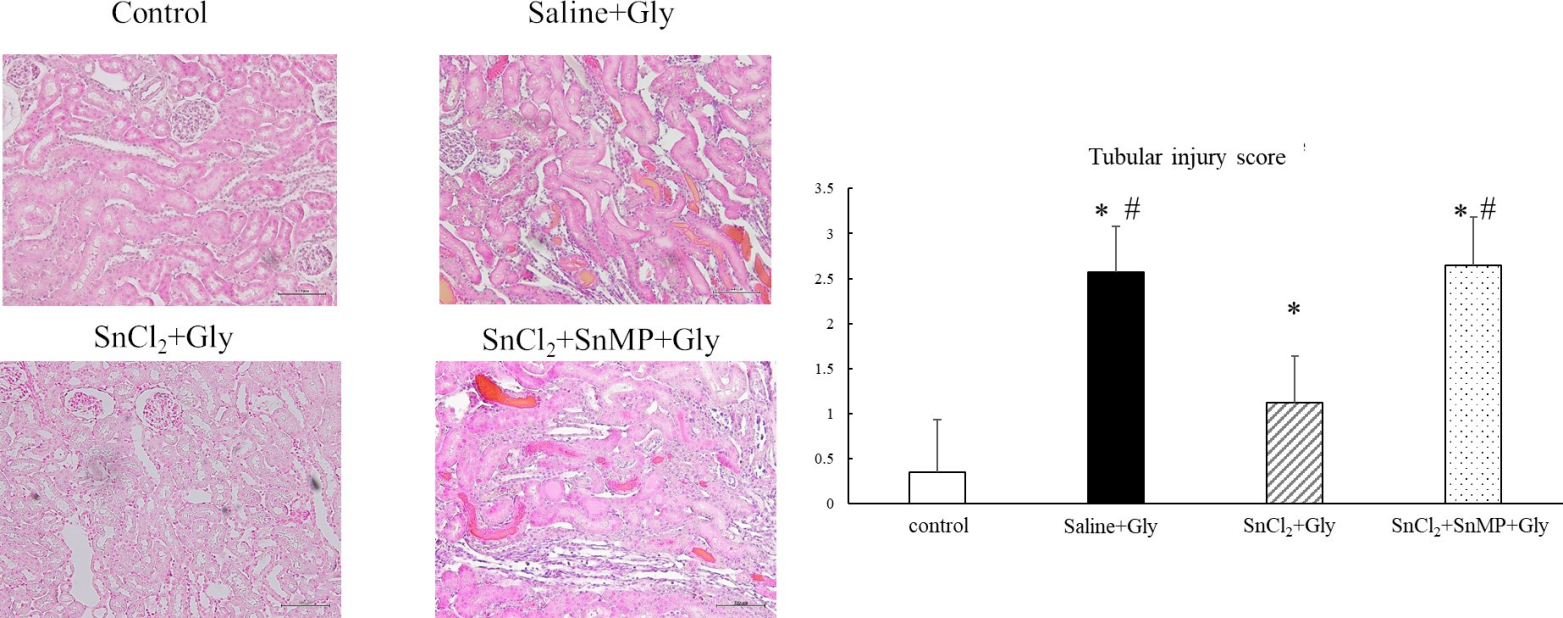
Effect of SnCl_2_ pretreatment on renal histopathological changes at 24 h after Gly treatment. Renal tissues were stained with HE and assessed using a light microscope. Representative images 24 h after Gly treatment (HE staining, original magnification × 200, scale bar = 100 μm). The severity of histopathological changes was evaluated using a tubular injury score. A total of 40 areas from a renal section of each group were assessed. The tubular injury was defined as tubular epithelial cell swelling, vacuolar degeneration, necrosis, and desquamation. Tubular injury score was graded by estimating the percentage of injured tubules as follows: 0, areas of injured tubules < 5%; 1, areas of injured tubules 5–25%; 2, areas of injured tubules 26–50%; 3, areas of injured tubules 51–75%; and 4, areas of injured tubules > 75%. The mean score for each kidney is provided, followed by the mean score for the group. Data are expressed as the mean ± SD (n = 4). * p < 0.05 vs control group; # p < 0.05 vs SnCl_2_+Gly group.

We also evaluated the effect of SnCl_2_ pretreatment on histopathological changes in renal tissues at 24 h after Gly treatment. Histological damages such as tubular epithelial cell swelling, vacuolar degeneration, necrosis, and desquamation were observed in the saline+Gly group. However, these histopathological changes were markedly improved with SnCl_2_ treatment, whereas SnMP treatment abrogated these improvements. Consistent with the histological findings, the tubular injury scores of the saline+Gly group were significantly greater than those of the control group; however, the SnCl_2_ treatment significantly reduced the score. Additional treatment with SnMP mitigated the protective effects of SnCl_2_ (Fig 5).

**Fig 6:**
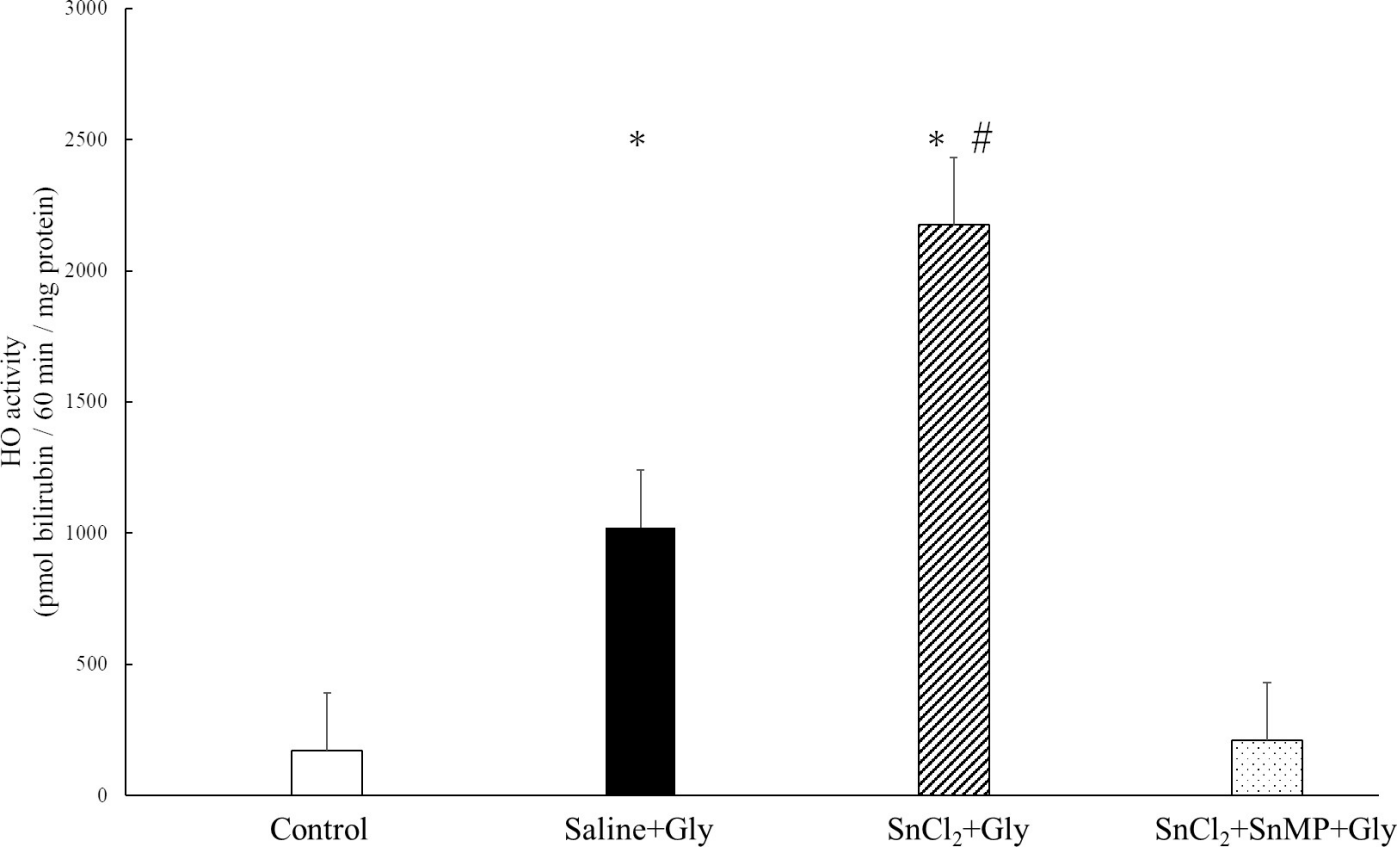
Effect of SnCl_2_ pretreatment on the renal HO activity at 24 h after Gly treatment. Renal tissues were collected 24 h after Gly treatment, and HO activity was assessed as described in Material and Methods. Data are expressed as the mean ± SD (n = 3–4). *p < 0.05 vs control group; #p < 0.05 vs saline+Gly group.

Furthermore, to confirm whether the protective effect of SnCl_2_ can be attributed to the enzymatic activity of HO-1, we measured renal HO activity after 24 h of Gly treatment in each group. While Gly treatment alone significantly increased renal HO activity when compared with the activity in the control group (control; 170 ± 109.16 vs saline+Gly; 1021.25 ± 132.81, pmol bilirubin/60 min/mg protein, respectively, p = 0.0492). Further treatment of SnCl_2_ robustly increased its activity in comparison to that of the saline+Gly group (saline+Gly;1021.25 ± 132.81 vs SnCl_2_+Gly; 2176.66 ± 1004.2 pmol bilirubin/60 min/mg protein, respectively, p = 0.0434). In contrast, additional treatment with SnMP suppressed the activity (Fig 6).

### Effect of SnCl_2_ pretreatment on the expression of ALAS1 mRNA in RM-AKI

**Fig 7:**
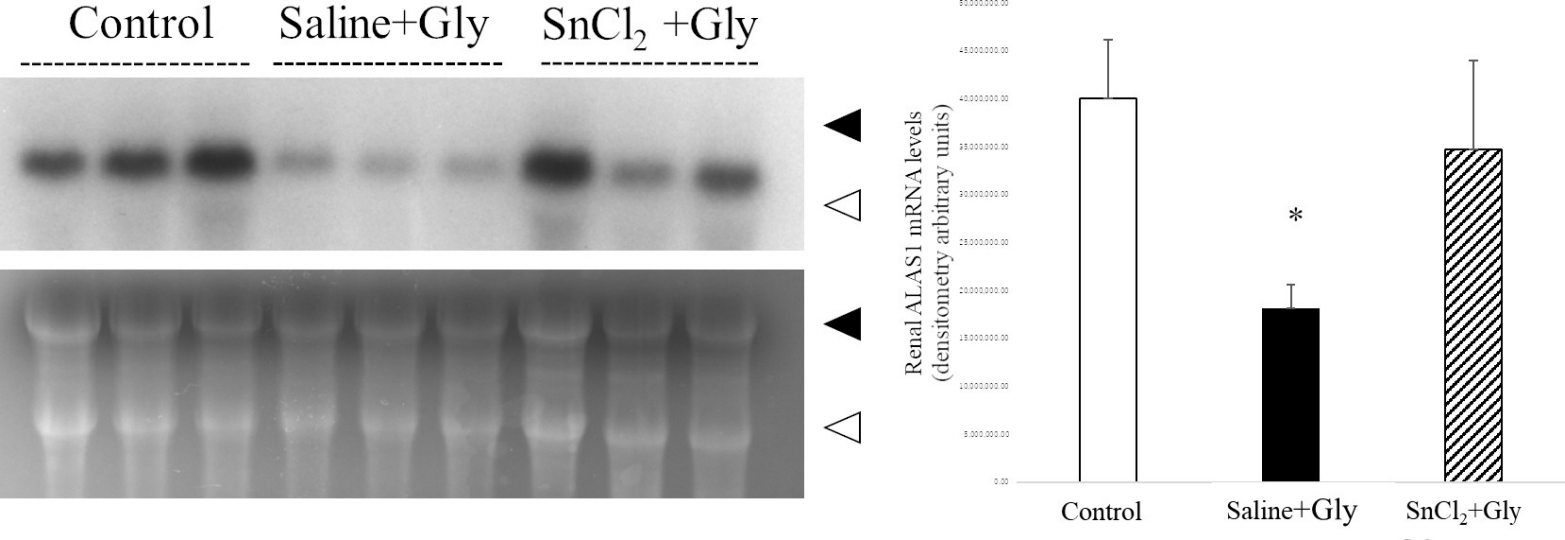
Expression of renal ALAS1 mRNA levels 3 h after Gly treatment. Rats pretreated with saline or SnCl_2_ (s.c.) were sacrificed 3 h after Gly treatment (10 mL/kg of 50% glycerol, i.m.), and renal tissues were collected for northern blot analysis. Control, untreated control rats; saline+Gly, saline-treated before Gly-treated rats; SnCl_2_+Gly, SnCl_2_-treated before Gly-treated rats. Ethidium bromide staining of the same RNA is shown as a loading control. Closed arrowhead, 28S ribosomal RNA; open arrowhead, 18S ribosomal RNA. ALAS1 mRNA expression levels are expressed as arbitrary densitometric units. Data are expressed as the mean ± SD. *p < 0.05 vs control group.

Next, to further clarify the mechanism behind the protective effect of HO-1 by SnCl_2_ treatment, we examined the effect of SnCl_2_ pretreatment on the expression of ALAS1, the rate-limiting enzyme of heme synthesis, which is known to be downregulated by heme. We previously reported that renal ALAS1 mRNA levels in rats treated with Gly alone showed a rapid decline at 1 h after treatment, reaching its nadir at 3 h, suggesting a rise in intracellular heme concentration by Gly treatment [11]. Accordingly, we examined the effect of SnCl_₂_ pretreatment on the expression of renal ALAS1 mRNA levels at 3 h after Gly treatment. While renal ALAS1 mRNA was significantly expressed in intact control animals, the levels in the saline+Gly group were markedly lower compared with those in the control group, as expected. However, their levels did not decrease in the SnCl_2_-pretreated group (Fig 7). These results suggest that SnCl_2_ pretreatment suppressed the increase in intracellular heme concentration induced by the Gly treatment.

### Effect of SnCl_2_ pretreatment on the expression of nuclear Bach1 protein in RM-AKI

**Fig 8:**
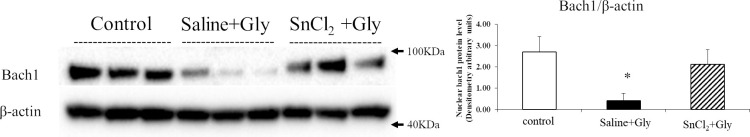
Renal nuclear Bach1 protein expression levels 3 h after Gly treatment. Rats pretreated with saline or SnCl_2_ (s.c.) were sacrificed 3 h after Gly treatment (10 mL/kg of 50% glycerol, i.m.), and renal tissues were collected for western blot analysis. Control, untreated control rats; saline+Gly, saline-treated before Gly-treated rats; SnCl_2_+Gly, SnCl_₂_-treated before Gly-treated rats. The densitometric ratio between Bach1 and β-actin was calculated. Data are expressed as the mean ± SD. *p < 0.05 vs control group.

Next, to elucidate the effect of SnCl_2_ pretreatment on the distribution of intracellular heme levels, we evaluated the effect of SnCl_2_ treatment on the protein expression of renal nuclear Bach1, a transcription repressor exported from the nucleus when it is bound by its ligand, heme. We previously showed that treatment of intact rats with Gly revealed a rapid decline in nuclear Bach1 protein levels, which reached the lowest level at 3 h, suggesting an increase in the nuclear heme concentration after Gly injection [11]. Thus, we evaluated the effect of SnCl_2_ pretreatment on the expression of renal nuclear Bach1 protein levels 3 h after Gly treatment. While renal Bach1 was significantly expressed in the control group, its levels in the saline-treated group were greatly reduced compared to those in the control group. In contrast, renal Bach1 levels in the SnCl_2_-treated group were almost identical to those in the control group (Fig 8). These findings suggest that SnCl_2_ prevented the increase in intra-nuclear heme concentration.

### Effect of SnCl_2_ administration in intact rats on serum creatinine, CPK, and ALT levels, and the expressions of HO-1, ALAS1, and nuclear Bach1 in the kidney

We administered SnCl_2_ subcutaneously to intact rats and examined changes in the serum levels of creatinine, as a measure of renal function, CPK, as a measure of muscle damage, and ALT, as a measure of hepatic function, respectively, at 1, 3, 6, 12, and 24 h after treatment. None of the indices changed significantly throughout the time course of the experiments (S1 Fig). We also evaluated the effect of SnCl_2_ administration in intact animals on the expression of HO-1 mRNA and protein, ALAS1 mRNA, and nuclear Bach1 protein in the kidney. SnCl_2_ treatment markedly increased HO-1 mRNA levels compared with levels in the control rats at 12 h (S2 Fig). HO-1 protein levels started to increase at 3 h and gradually increased in a time-dependent manner, and then reached a maximum at 12 h after the treatment, which was maintained by 24 h (S3 Fig). The levels of ALAS1 mRNA and nuclear Bach1 protein were not significantly influenced by SnCl_2_ treatment at 1, 3, 6, 12, and 24 h (S4 and S5 Figs). These results suggest that SnCl_2_ treatment itself induced HO-1 in the kidney, at least in part, mediated through a mechanism other than the increase in intracellular heme levels without causing major adverse effects in rats.

## Discussion

We have demonstrated for the first time that SnCl_2_ pretreatment ameliorates RM-AKI induced upon injecting glycerol into the hind limbs of rats. This could be attributed to the early, enhanced, and persistent induction of HO-1 expression by SnCl_2_ in the kidney as SnCl_2_ is a kidney-specific inducer of HO-1 [9]. In demonstrating our hypothesis, our findings showed that the inhibition of renal HO activity by SnMP, a specific competitive inhibitor of the enzyme, entirely abolished the beneficial effect of HO-1 induction. We also showed that renal expression of ALAS1 and Bach1 (at mRNA and nuclear protein levels, respectively) in SnCl_2_-pretreated animals was maintained almost to the same extent as that in naïve animals, while this expression was significantly suppressed in saline-pretreated animals. ALAS1 is downregulated by heme [17,18], and Bach1 is exported from the nucleus in complexes that contain increased intracellular heme content [19,20]. Thus, our findings suggest that overexpression of HO-1, the rate-limiting enzyme in heme catabolism, by SnCl_2_ pretreatment confers protection against rat RM-AKI as it degrades the excess amount of intracellular free heme, a pro-oxidant released from myoglobin.

Myoglobin-derived oxygen free radicals have been implicated in the pathogenesis of RM-AKI. In accordance with this hypothesis, it has been reported that oxidative stress plays a crucial role in the pathogenesis of RM-AKI by validating the increase in the renal concentration of 8-OHdG (8-hydroxy-2’-deoxyguanosine), an indicator of lipid peroxidation [21,22] and malondialdehyde (MDA), an indicator of oxidative stress, and a decrease in the renal levels of superoxide dismutase (SOD), an antioxidant enzyme [23–25]. In contrast, HO-1 has been reported to play a protective role in various animal models of oxidative stress-induced organ injury, including RM-AKI, due to its antioxidant property [6]. Nath et al. reported that HO-1 induction by hemoglobin has a protective effect against RM-AKI [26]. They also showed that the induction of HO-1 is an indispensable response in protecting against RM-AKI in HO-1 knockout mice [4]. They also proposed that the protective effects of HO-1 can be attributed to heme degradation products such as CO and biliverdin, both of which have anti-inflammatory and antioxidative properties [8]. We reported that renal HO-1 was induced in a rat model of ischemic acute renal failure generated in response to renal ischemia/reperfusion (I/R), which leads to oxidative stress in the kidney, and played a protective role against I/R kidney injury [27]. Furthermore, we showed that kidney-specific induction of HO-1 by SnCl_2_ pretreatment prevented ischemic acute kidney injury [10]. In the present study, pretreatment with SnCl_2_ elevated HO-1 protein significantly and ameliorated renal injury significantly, both in blood biochemical findings and in histological findings, and SnMP administration counteracted the effect. The findings suggest that SnCl_2_ pretreatment confers protection against renal oxidative damage in RM-AKI due to its ability to induce the expression of renal HO-1.

During RM-AKI, destabilization of myoglobin derived from the injured striated muscle can lead to a significant increase in cellular free heme levels [1]. “Free heme” represents newly produced heme that is not complexed with its apohemoprotein or free heme released from a hemoprotein but not yet cleaved by HO. While heme is required as a prosthetic group of myoglobin, excess amount of free heme released from damaged myoglobin is toxic to the tubular epithelial cells because it can intercalate into the phospholipid bimolecular membrane to generate oxygen free radicals as lipophilic iron, ultimately leading to cellular injury [28]. Thus, in a quiescent state, cellular free heme levels are maintained at extremely low levels (approximately 100 nM) through two opposing enzymes, namely, ALAS1, the rate-limiting enzyme in heme synthesis, which is downregulated by heme, and HO-1, the rate-limiting enzyme in heme catabolism, which is upregulated by heme [28]. We have previously demonstrated that in the rat RM-AKI model—which is the same model used in this study—following glycerol injection, there was a significant increase in endogenous HO-1 expression and a rapid and robust decrease in ALAS1 expression, suggesting a significant improvement in the renal intracellular free-heme concentration after glycerol treatment [11]. In the present study, ALAS1 mRNA levels in saline-pretreated RM-AKI rats decreased significantly 3 h after Gly treatment; however, the level was maintained in SnCl_2_-pretreated RM-AKI rats. The results suggest that early and enhanced SnCl_2_-induced HO-1 degrades excess free heme, a pro-oxidant derived from destabilized myoglobin, thereby conferring protection against oxidative tissue injury in RM-AKI.

HO-1 is induced not only by its substrate heme but can also be induced by other substrates, including metals, cytokines, and oxidative stress [2,3]. It has been reported that heme induces HO-1 through the Maf recognition element (MARE) in the promoter region of the *ho-1* gene in cultured cells [20]. Under baseline conditions, Bach1, a heme responsive transcription factor, binds to a small Maf protein to form a heterodimer that binds to the MARE in the promoter region of *ho-1*, leading to the repression of transcription of HO-1. However, in the presence of excessive heme, the binding of heme to Bach1 results in its dissociation from MARE, its subsequent translocation into the cytoplasm, and further ubiquitination and degradation [19,20,29–31]. Dissociation of Bach1 allows Maf to dimerize with another transcription factor, NF-E2-related factor 2 (Nrf2), which allows transcriptional activation of HO-1. In addition, in an *in vitro* study, heavy metals such as Cd and Co have been reported to induce HO-1 mediated through the metal responsive element (MRE), which is also located in the promoter region of *the ho-1* gene. We have previously demonstrated that following glycerol treatment, in a RM-AKI rat model, nuclear Bach1 levels rapidly and significantly decreased 3 h after treatment, which was preceded by the induction of HO-1 mRNA expression, suggesting that nuclear Bach1 was displaced from MARE and exported from the nucleus by directly binding to heme released from myoglobin, ultimately leading to HO-1 induction [11]. In the present study, nuclear Bach1 protein levels in saline-pretreated RM-AKI rats showed significant decreases 3 h after Gly treatment; however, the levels in the SnCl2-pretreatment RM-AKI rats were not altered. Taken together, we speculate that SnCl_2_ administration prior to glycerol treatment caused HO-1 induction, possibly via the metal-responsive element that overwhelmed the heme-mediated endogenous HO-1 induction, leading to early and persistent HO-1 expression in SnCl_2_-pretreated RM-AKI animals. Although we did not directly show that SnCl_2_ induced HO-1 via metal responsive elements in rats, the fact that administration of SnCl_2_ in intact animals induced HO-1 mRNA and protein expression in the kidney without influencing the expression of ALAS1 mRNA and nuclear Bach1 protein could support our hypothesis.

Numerous reports have indicated that overexpression of HO-1 has beneficial effects against oxidative tissue injury [5,6,8]. However, the enhanced HO-1 expression may have adverse effects. Higher levels of HO activity have been shown to reduce the concentration of other heme proteins, such as cytochrome P450 enzymes, that are necessary for cell viability and release of labile iron, which also catalyzes free radical formation if insufficiently sequestered [3,32]. An adequate amount of cellular heme is also required to fulfill important cellular signaling pathways, including gene expression, proliferation, cell function, and differentiation [3,33–35]. Thus, the prolonged overexpression of HO-1, for instance by gene transfer, is likely to have deleterious effects through enhanced heme degradation. Systemic induction of HO-1 by primary HO-1 inducers such as hemoglobin and hemin at appropriate doses has also been reported to confer protection against various animal models of oxidative tissue injury [24,36]. However, when considering its application in clinical practice, administration of these primary inducers can cause further debt of oxidative stress by increasing intracellular heme levels, a pro-oxidant, prior to the induction of HO-1 in the target organ. In addition, systemic induction of HO-1 may cause fluctuation of heme metabolism to some extent in various organs other than the target organ, both of which could induce an untoward response. Taken together, it is thought that the desirable method of HO-1 induction against oxidative tissue injury, especially heme-mediated oxidative tissue injury including RM-AKI, is the administration of pharmacological substances that induce an appropriate amount of HO-1 in a target organ-specific manner without causing a transient increase in intracellular heme levels. We reported that SnCl_2_ induced HO-1 in a kidney-specific manner and prevented ischemic acute kidney injury by degrading excess intracellular heme. Moreover, as we have mentioned earlier, SnCl_2_ is considered to induce HO-1 expression, possibly through a metal-responsive element, without resulting in a transient increase in intracellular heme levels. Thus, SnCl_2_ has properties that can be applied as a pharmacological inducer of HO-1 in RM-AKI. However, SnCl_2_ is known to be toxic to humans. Thus, pharmacological inducers exhibiting these favorable characteristics and without adverse effects should be pursued in future clinical applications.

The present study has several limitations. First, we treated the RM-AKI rats with SnCl_2_ at a dose of 10 mg/kg. However, we should have examined the dose-response relationship of the protective effect of SnCl_2_ to underscore the beneficial effect of SnCl_2_ on RM-AKI, as well as to determine the minimum and optimal dose of SnCl_2_ to reveal its protective effect on RM-AKI because it is toxic at high doses, even in rats. Second, we administered SnCl_2_ 24 h prior to glycerol injection. To employ HO-1 inducers in clinical practice, they have to be administered after noxious insult. Thus, experiments to investigate the therapeutic effect of SnCl_2_ on RM-AKI remain to be elucidated in future studies. Third, the free heme concentration in the renal tissue of our rat RM-AKI model was not directly measured. The usual levels of free heme in normal cells is too low (<10^−9^ M) to be assessed using conventional methods, although we have developed a novel heme sensor using fluorescently labeled HO-1 that enables us to measure free heme concentration in the rat liver [37]. Moreover, it has been reported that the microsomal heme concentration in the kidney is almost one-third of that in the liver [10,15], suggesting that free heme concentration is significantly lower in the kidney than in the liver. Thus, we estimated the suppression of the increase in renal free heme concentration by SnCl_2_ in RM-AKI animals as judged by the significantly higher expression of ALAS1 mRNA and nuclear Bach1 protein than those in saline-treated RM-AKI animals.

## Supporting information

S1 Checklist Our willingness to comply with the ARRIVE guidelines. (PDF)

S1 Fig Changes in serum biochemical parameters of saline or SnCl_2_ pretreated animals. Crea, creatinine. CPK, creatinine phosphokinase. ALT, alanine aminotransferase. *p < 0.05, vs control group.
(TIF)

S2 Fig Expression of renal HO-1 mRNA levels 12 h after saline or SnCl_2_ treatment (s.c.). Saline, saline-treated animals; SnCl_2_, SnCl_2_ treated animals. *p < 0.05, vs saline group.
(TIF)

S3 Fig Changes in the expression of renal HO-1 protein levels after SnCl_2_ treatment (s.c.). (TIF)

S4 Fig Changes in the expression of renal ALAS1 mRNA levels after SnCl_2_ treatment (s.c.). (TIF)

S5 Fig Changes in the expression of renal nuclear Bach1 protein levels after SnCl_2_ treatment (s.c.). (TIF)

S1 Raw images Original images for blots and gels. (PDF)
