# Supplementary figures and images for: Protective effect of tin chloride on rhabdomyolysis-induced acute kidney injury in rats
Source: PLoS One. 2022 Mar 16;17(3):e0265512. doi: 10.1371/journal.pone.0265512 (PMC8926186; doi:10.1371/journal.pone.0265512)

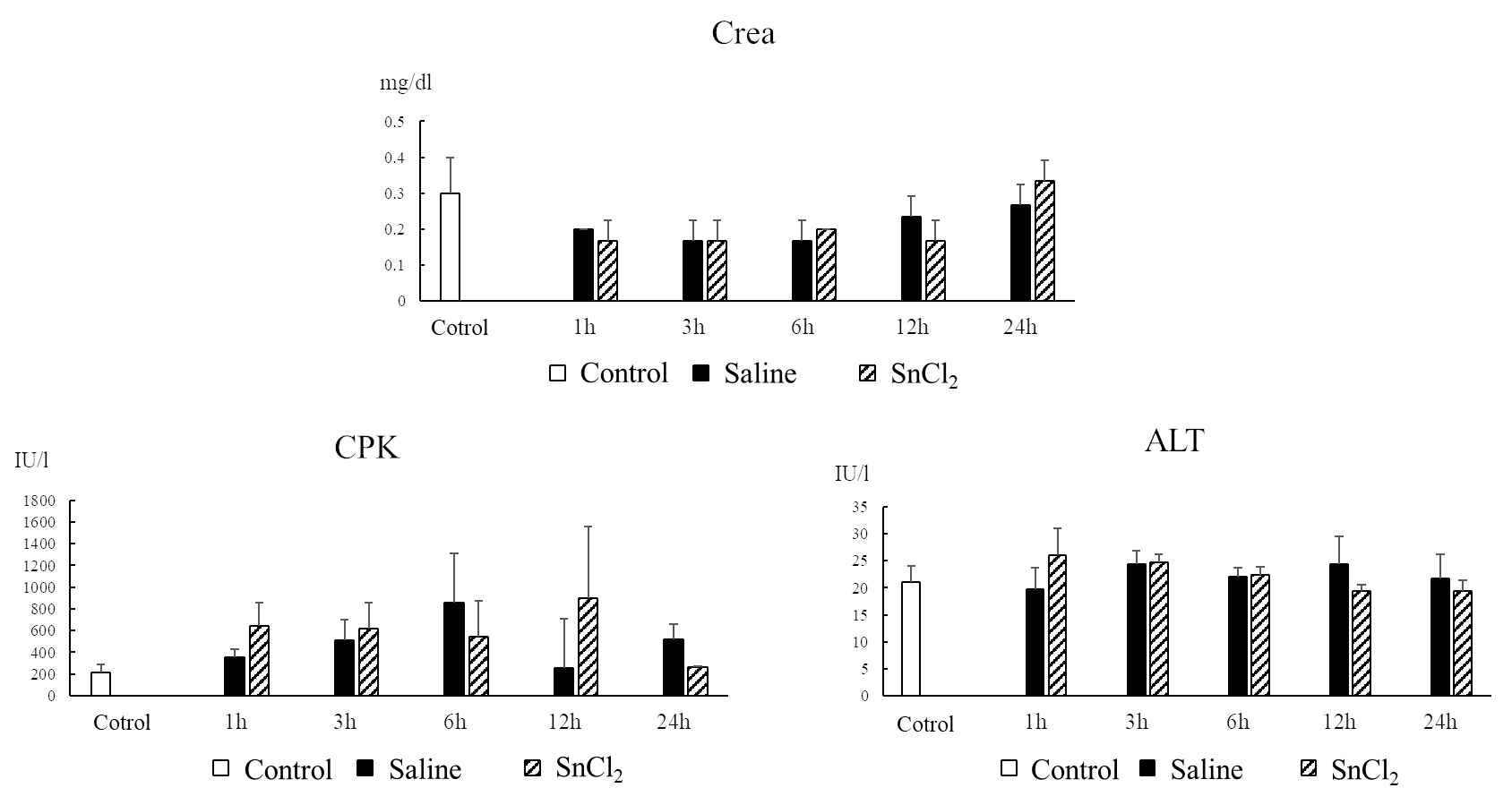

Supplement: S1 Fig — Crea, creatinine. CPK, creatinine phosphokinase. ALT, alanine aminotransferase. *p < 0.05, vs control group. (TIF) [file pone.0265512.s002.tif]

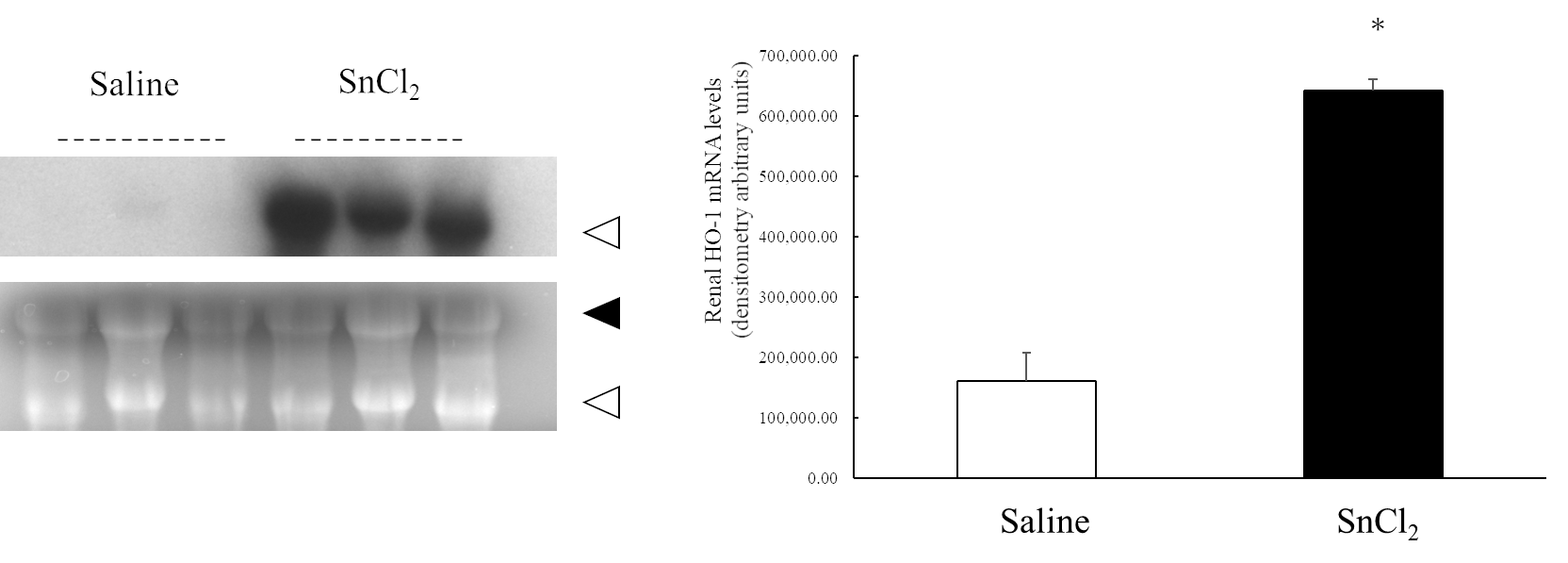

Supplement: S2 Fig — Saline, saline-treated animals; SnCl2, SnCl2 treated animals. *p < 0.05, vs saline group. (TIF) [file pone.0265512.s003.tif]

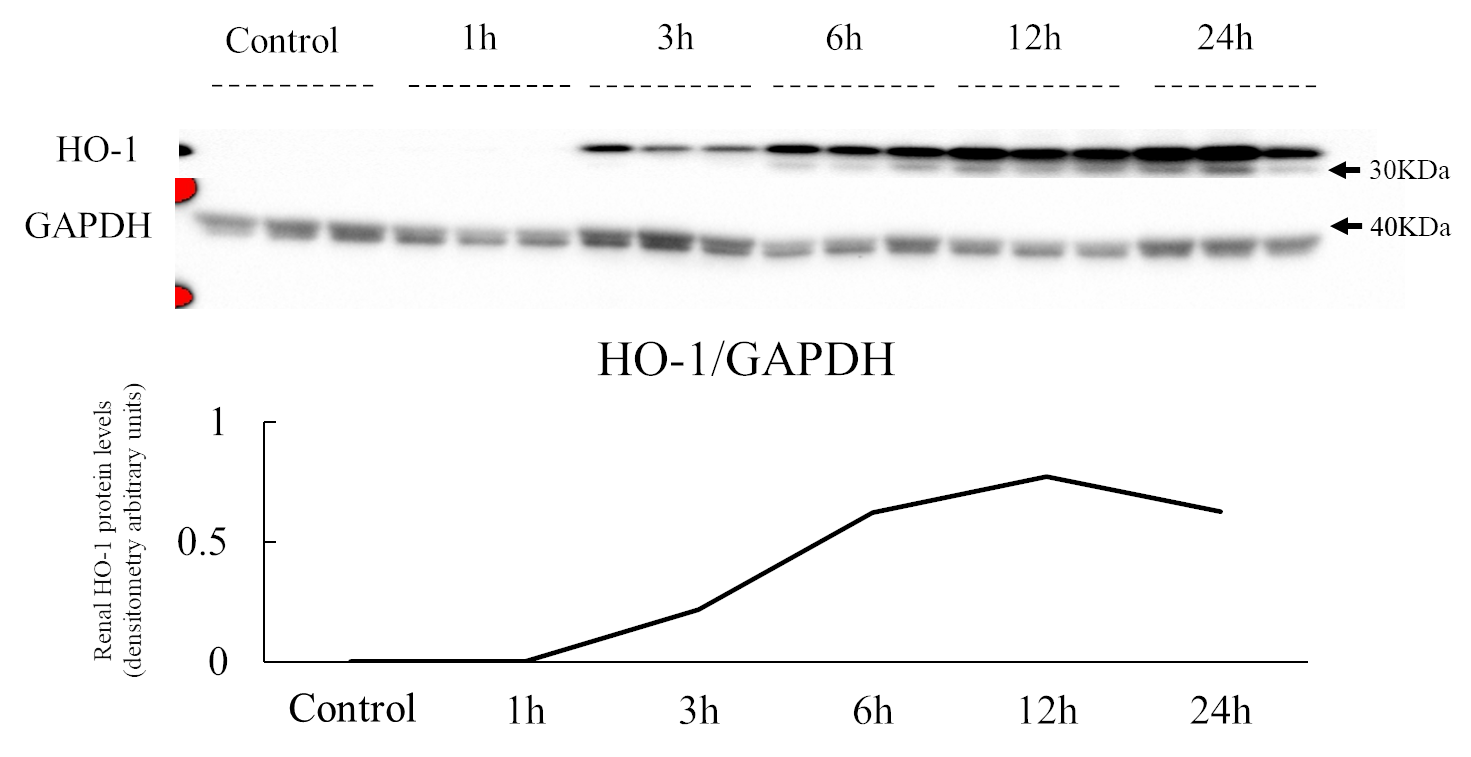

Supplement: S3 Fig — (TIF) [file pone.0265512.s004.tif]

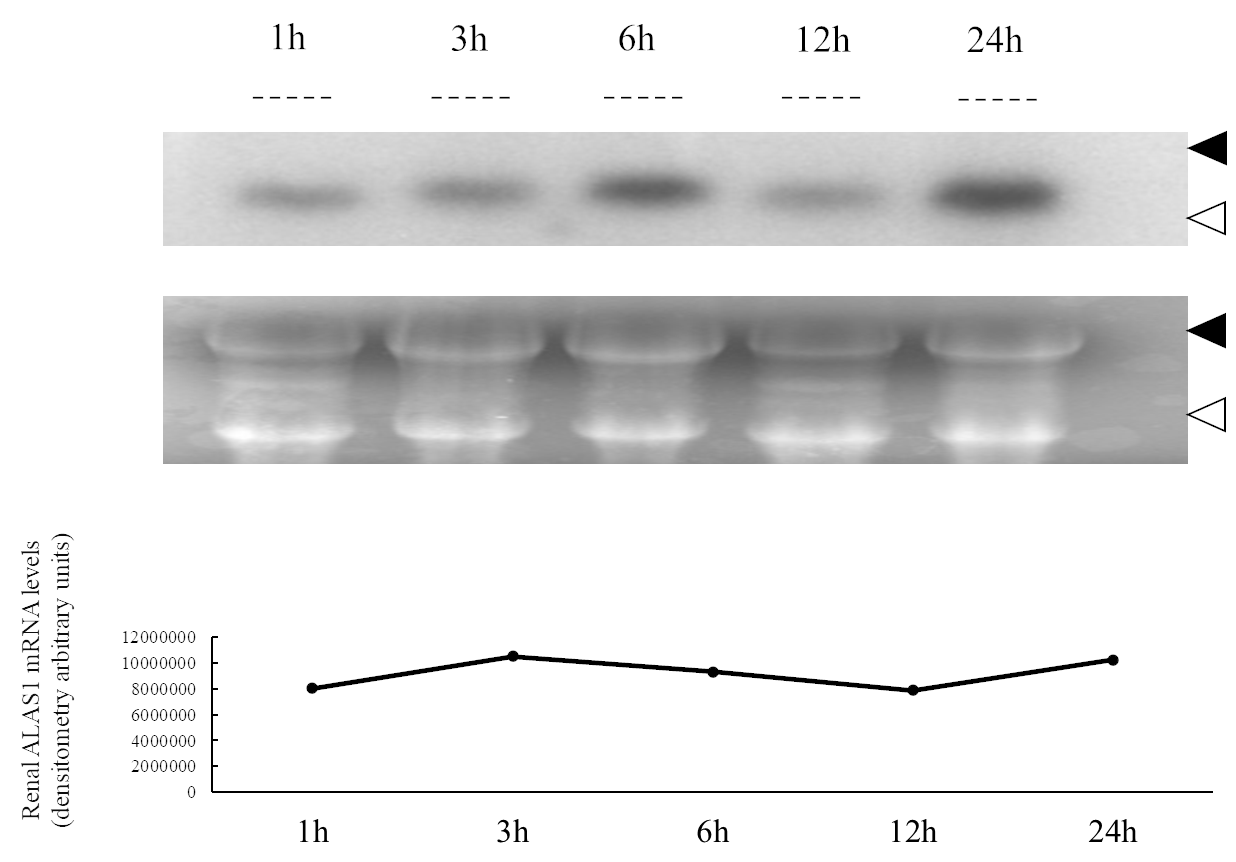

Supplement: S4 Fig — (TIF) [file pone.0265512.s005.tif]

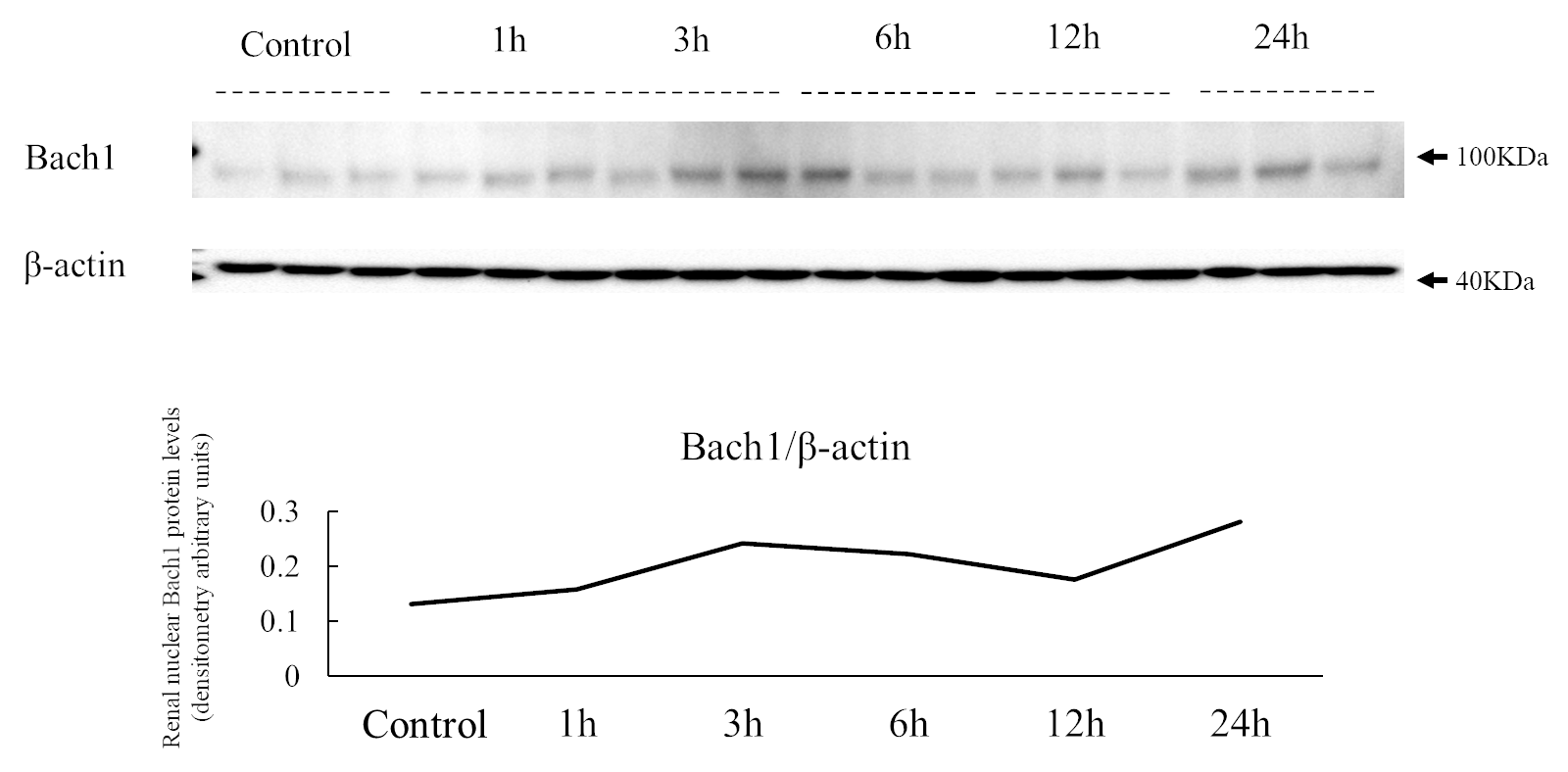

Supplement: S5 Fig — (TIF) [file pone.0265512.s006.tif]
