# Supplementary material for: Protective effect of tin chloride on rhabdomyolysis-induced acute kidney injury in rats
Source: PLoS One. 2022 Mar 16;17(3):e0265512. doi: 10.1371/journal.pone.0265512 (PMC8926186; doi:10.1371/journal.pone.0265512)
Supplement: S1 Raw images — (PDF) [file pone.0265512.s007.pdf]

Fig2 raw image

The samples were loaded in the order of their numbers.
In the figure 1-12 are shown.

Antigen-antibody complexes were stained with Clarity Western ECL Substrate (Bio-Rad) and visualization was performed using an image scanner (ChemiDoc XRS Plus Imaging System, Bio-Rad).

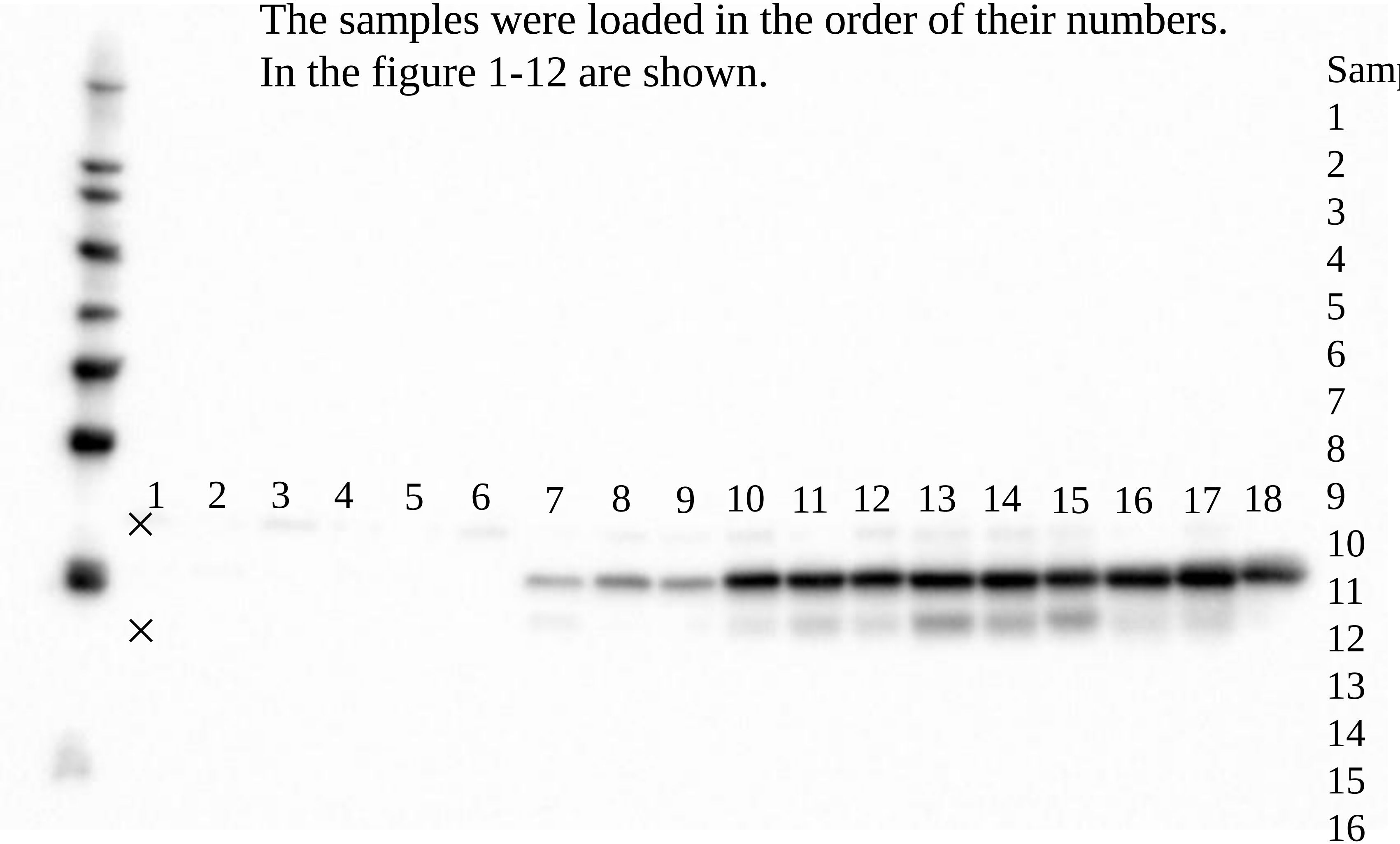

Sample ID	
1	2015051901
2	2015051902
3	2015051903
4	2018032801
5	2018032802
6	2018032803
7	2018022807
8	2018022808
9	2018022809
10	2018042501
11	2018042502
12	2018042503
13	2018032807
14	2018032808
15	2018032809
16	2017110701
17	2017110702
18	2017110703

Fig2 raw image

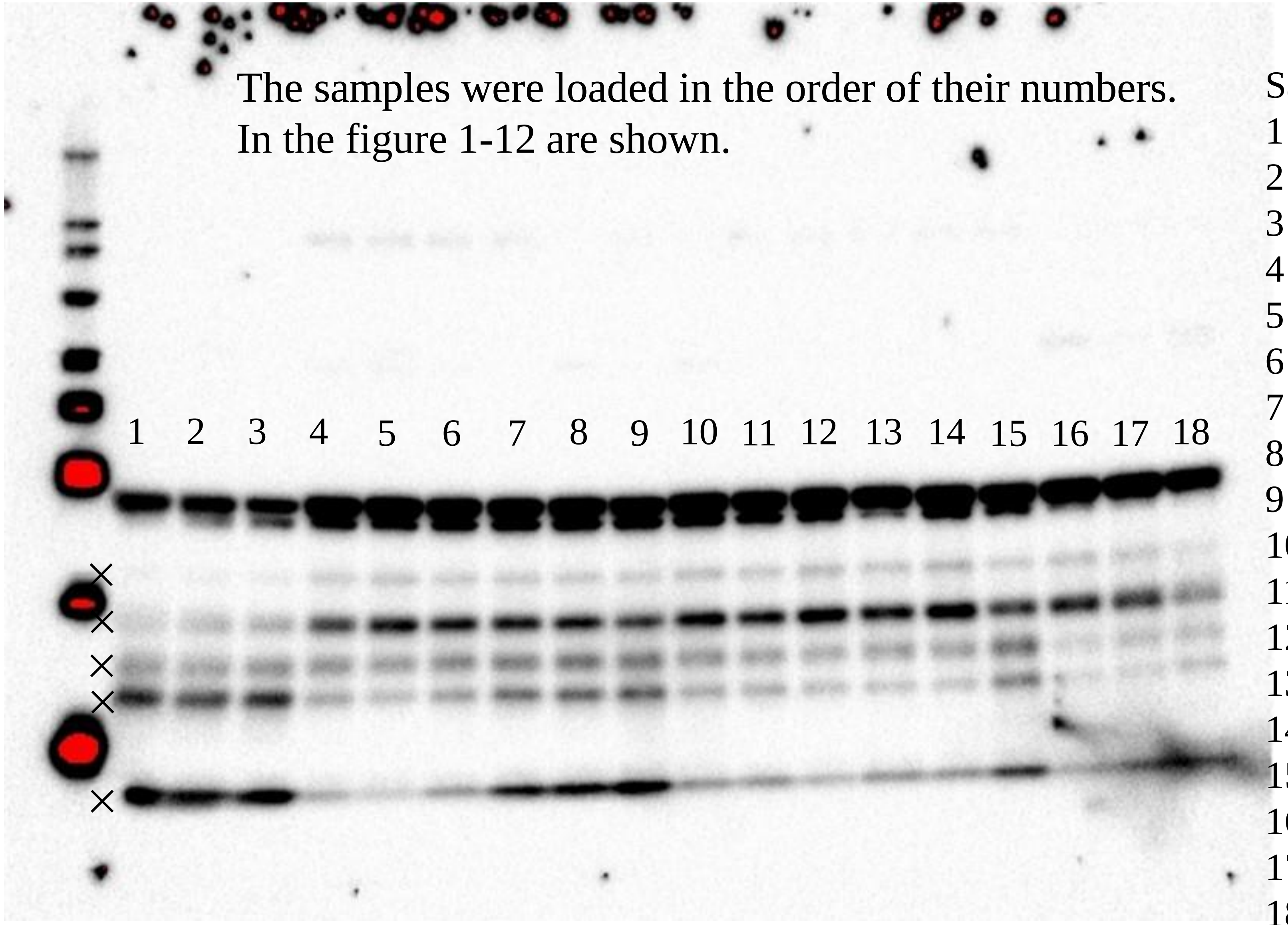

Antigen-antibody complexes were stained with Clarity Western ECL Substrate (Bio-Rad) and visualization was performed using an image scanner (ChemiDoc XRS Plus Imaging System, Bio-Rad).

Sample ID	
1	2015051901
2	2015051902
3	2015051903
4	2018032801
5	2018032802
6	2018032803
7	2018022807
8	2018022808
9	2018022809
10	2018042501
11	2018042502
12	2018042503
13	2018032807
14	2018032808
15	2018032809
16	2017110701
17	2017110702
18	2017110703

Fig2 raw image

The samples were loaded in the order of their numbers.
In the figure 1-12 are shown.

Antigen-antibody complexes were stained with Clarity Western ECL Substrate (Bio-Rad) and visualization was performed using an image scanner (ChemiDoc XRS Plus Imaging System, Bio-Rad).

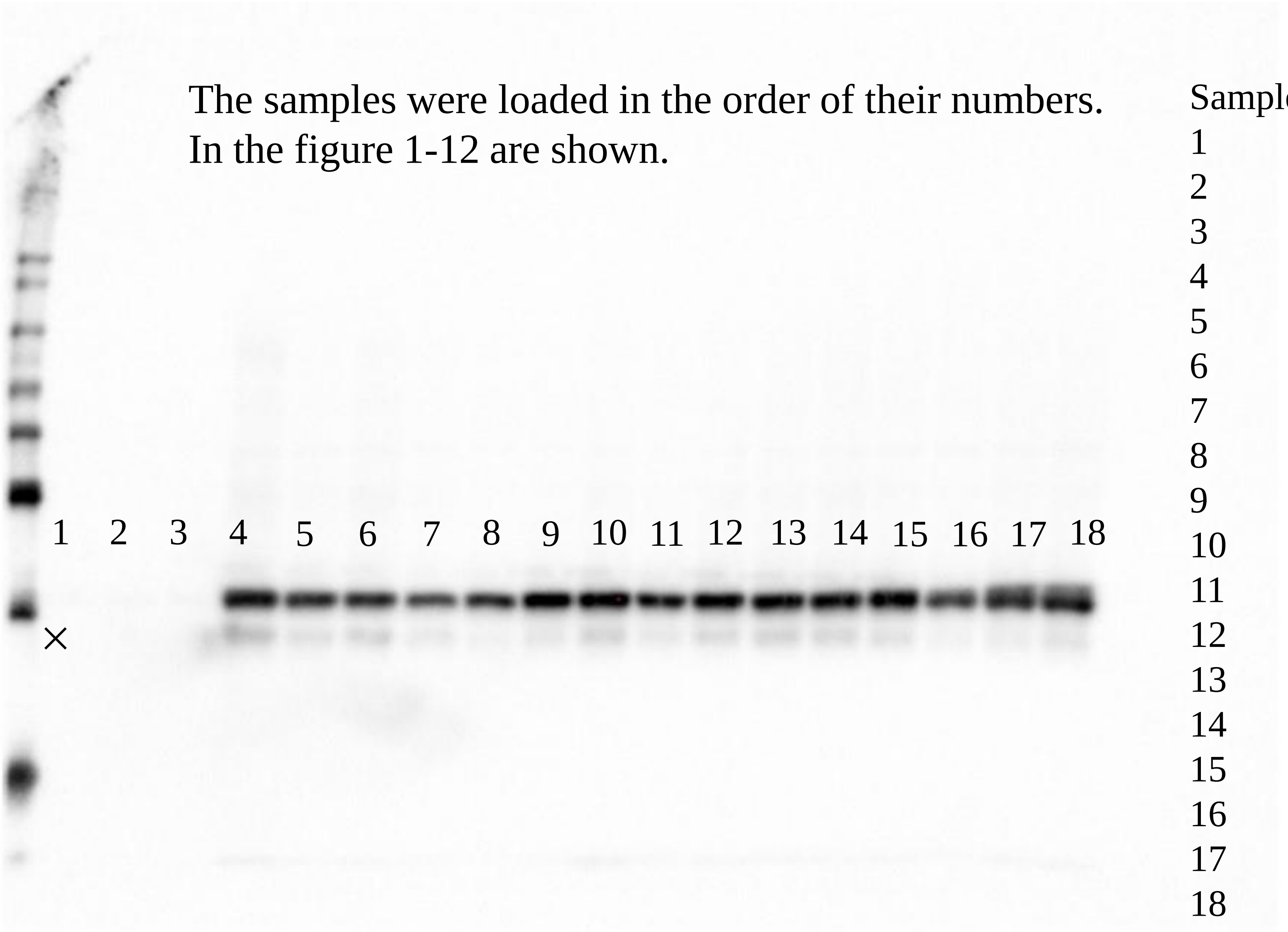

Sample ID	
1	2015051901
2	2015051902
3	2015051903
4	2018032804
5	2018032805
6	2018032806
7	2018022810
8	2018022811
9	2018022812
10	2018042504
11	2018042505
12	2018042506
13	2018032810
14	2018032811
15	2018032812
16	2017110704
17	2017110705
18	2017110706

Fig2 raw image

The samples were loaded in the order of their numbers.
In the figure 1-12 are shown.

Antigen-antibody complexes were stained with Clarity Western ECL Substrate (Bio-Rad) and visualization was performed using an image scanner (ChemiDoc XRS Plus Imaging System, Bio-Rad).

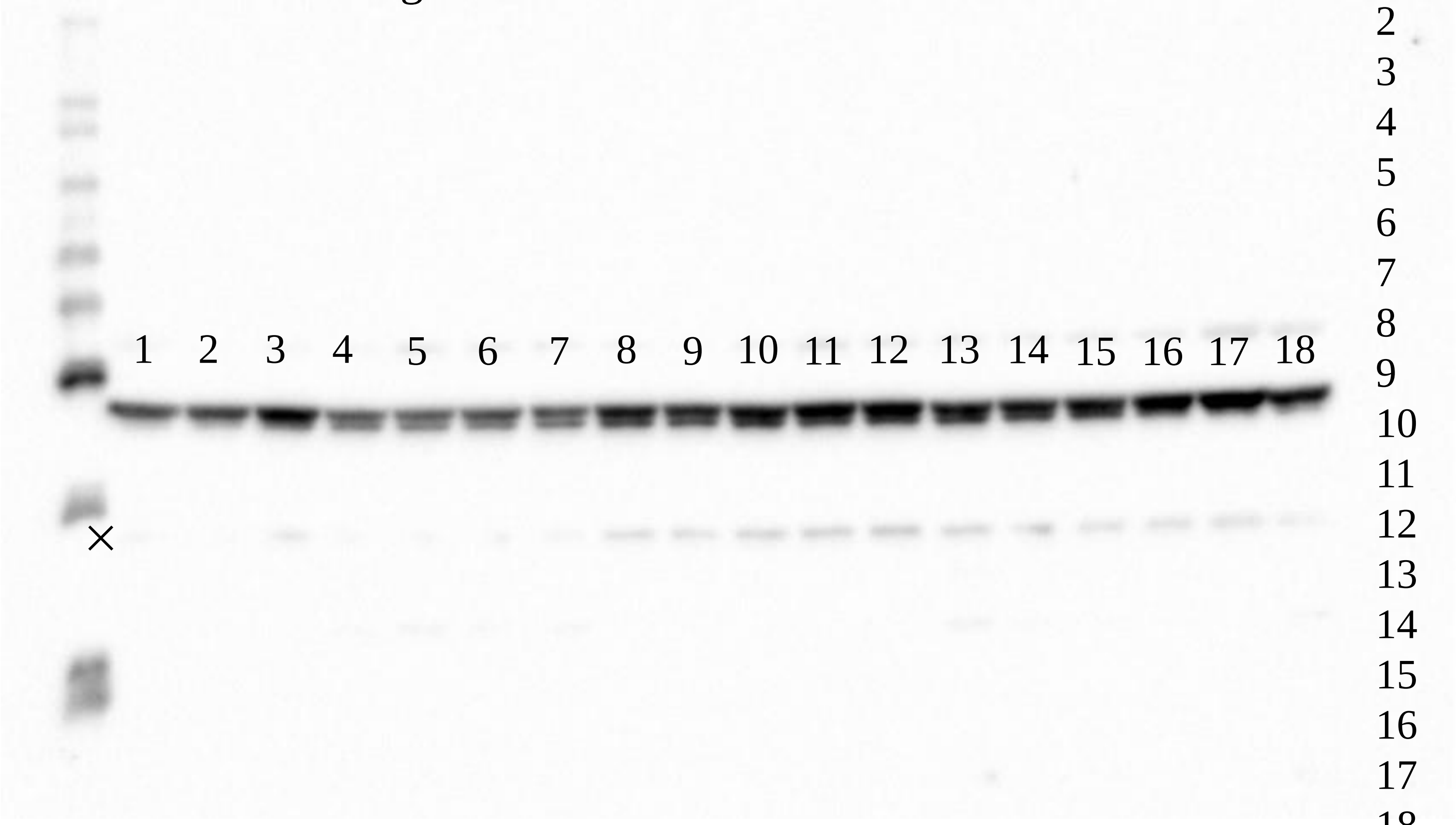

Sample ID	
1	2015051901
2	2015051902
3	2015051903
4	2018032804
5	2018032805
6	2018032806
7	2018022810
8	2018022811
9	2018022812
10	2018042504
11	2018042505
12	2018042506
13	2018032810
14	2018032811
15	2018032812
16	2017110704
17	2017110705
18	2017110706

Fig3 raw image

The samples were loaded in the order of their numbers.

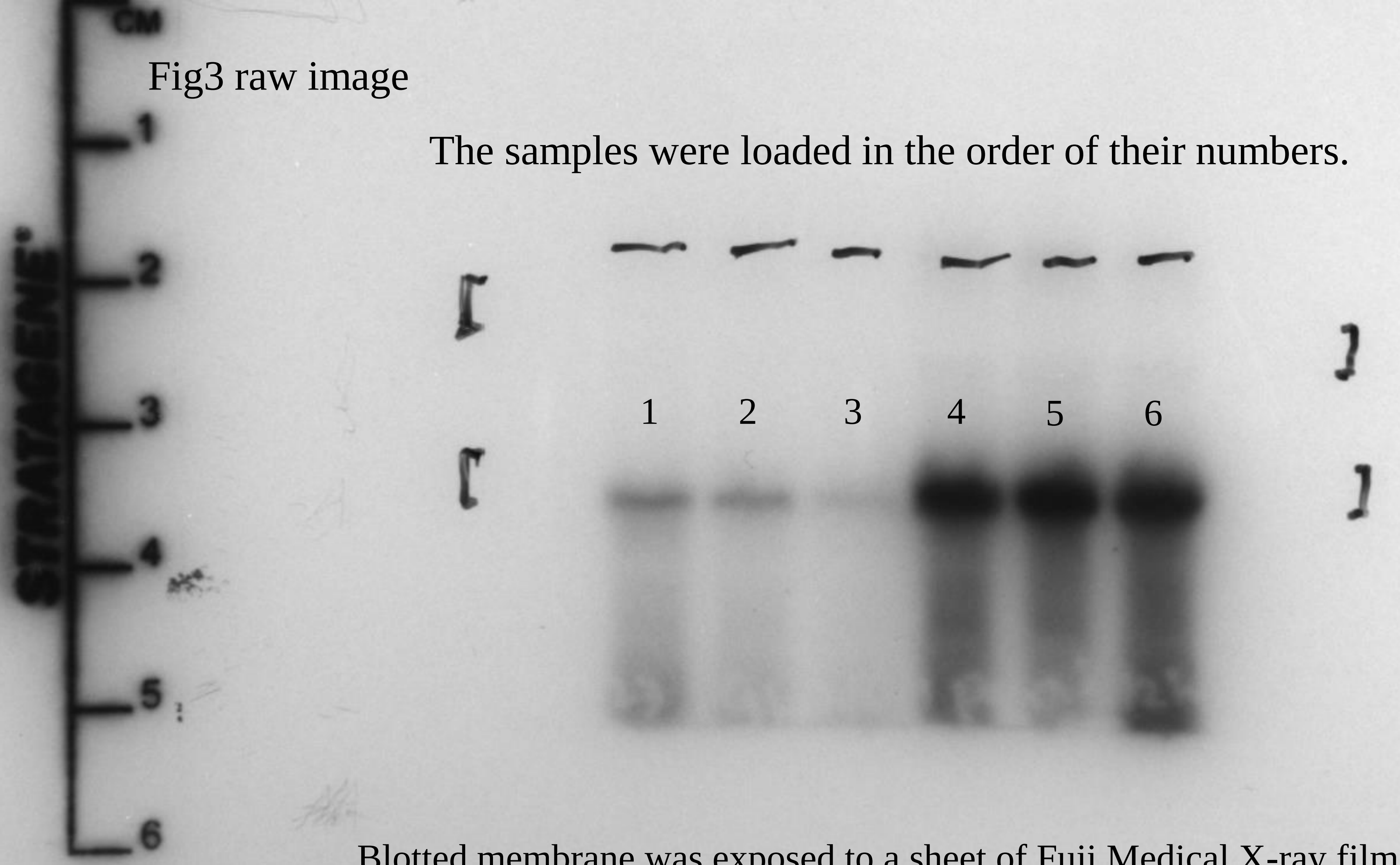

Sample ID	
1	2017082104
2	2017082105
3	2017082106
4	2018042504
5	2018042506
6	2018042507

Blotted membrane was exposed to a sheet of Fuji Medical X-ray film (Fujifilm Co., Tokyo, Japan) with an intensifying screen at −80 °C.

The samples were loaded in the order of their numbers.

Fig3 raw image

Signals corresponding to the target mRNA on the film and 18S ribosomal RNA bands visualized on the gel were estimated using an image scanner (ChemiDoc XRS Plus Image Processing System, Bio-Rad, USA) and image analysis software (Image Lab™ version 5.0; Bio-Rad Laboratories).

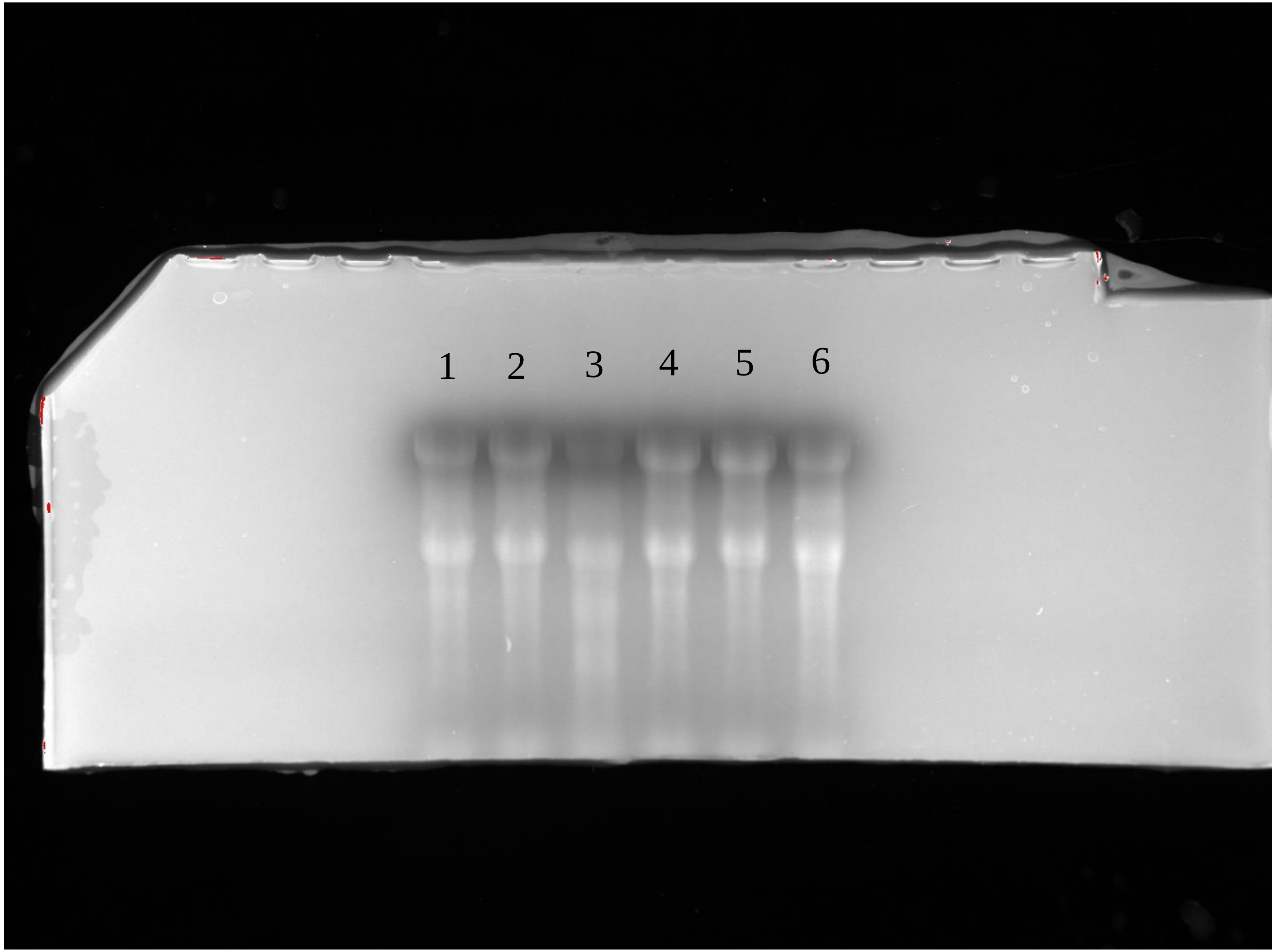

Sample ID	
1	2017082104
2	2017082105
3	2017082106
4	2018042504
5	2018042506
6	2018042507

Fig7 raw image

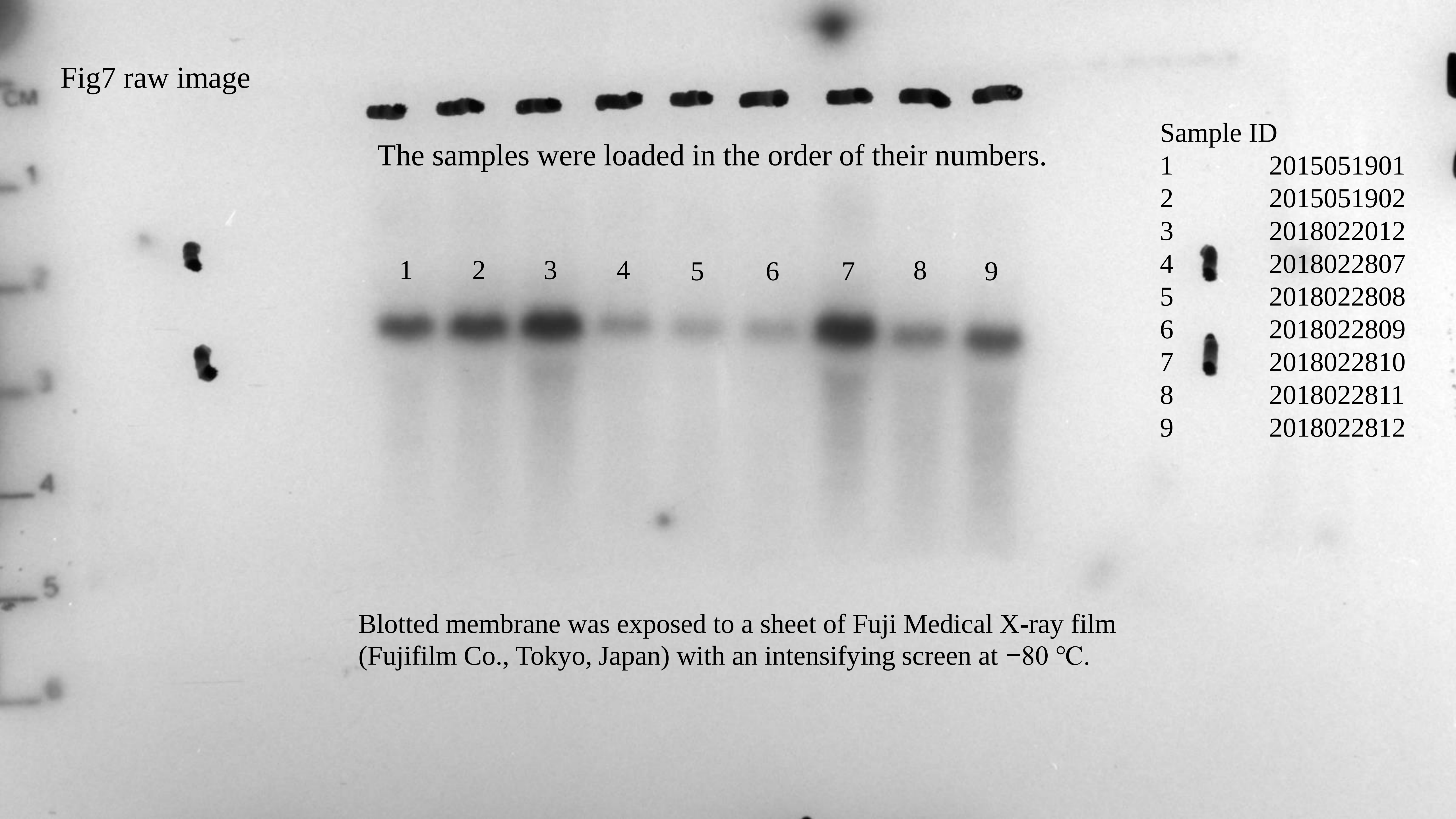

The samples were loaded in the order of their numbers.

Sample ID	
1	2015051901
2	2015051902
3	2018022012
4	2018022807
5	2018022808
6	2018022809
7	2018022810
8	2018022811
9	2018022812

Blotted membrane was exposed to a sheet of Fuji Medical X-ray film (Fujifilm Co., Tokyo, Japan) with an intensifying screen at −80 °C.

Fig7 raw image

Signals corresponding to the target mRNA on the film and 18S ribosomal RNA bands visualized on the gel were estimated using an image scanner (ChemiDoc XRS Plus Image Processing System, Bio-Rad, USA) and image analysis software (Image Lab™ version 5.0; Bio-Rad Laboratories).

The samples were loaded in the order of their numbers.

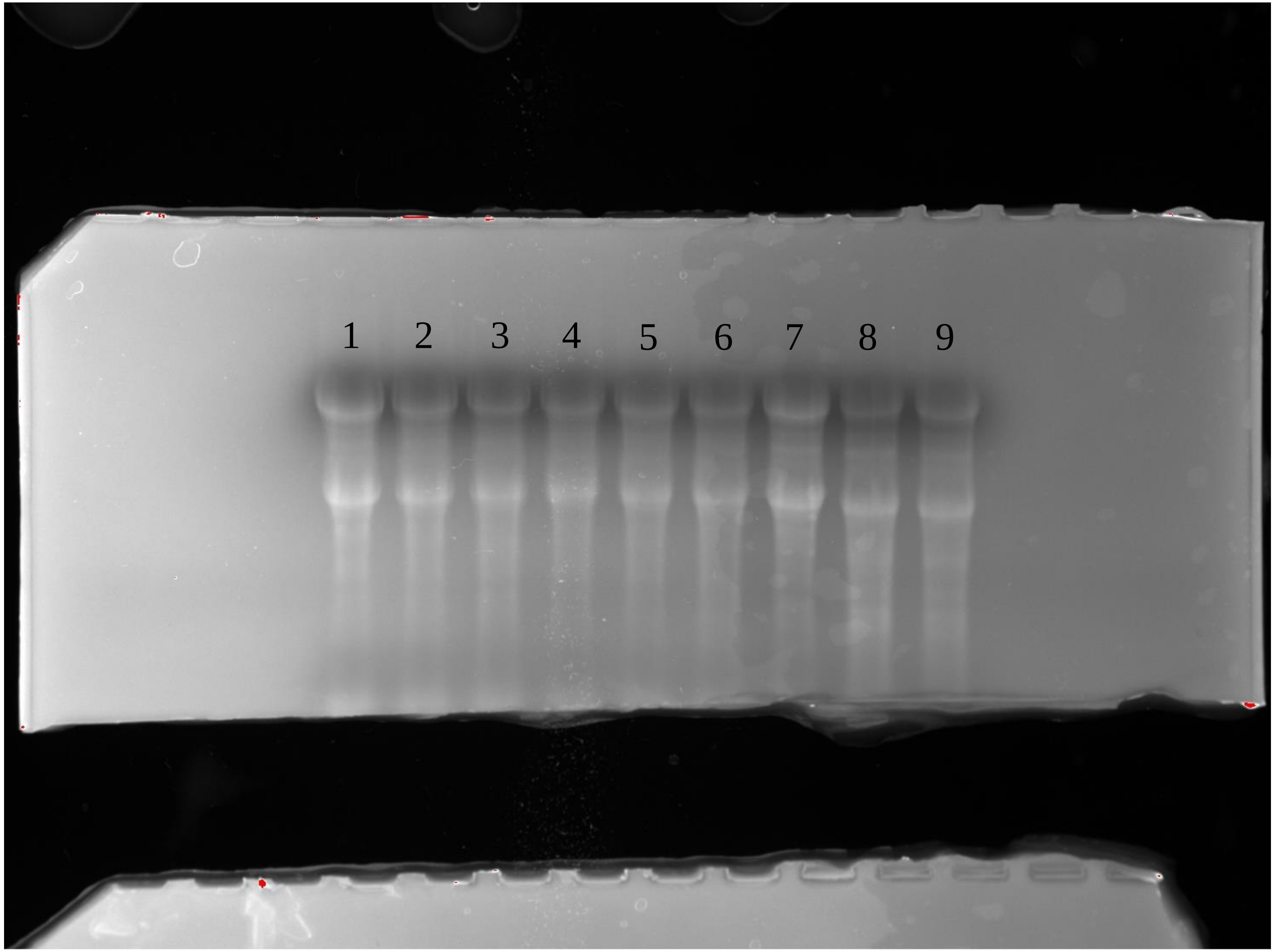

Sample ID	
1	2015051901
2	2015051902
3	2018022012
4	2018022807
5	2018022808
6	2018022809
7	2018022810
8	2018022811
9	2018022812

Fig8 raw image

The samples were loaded in the order of their numbers.

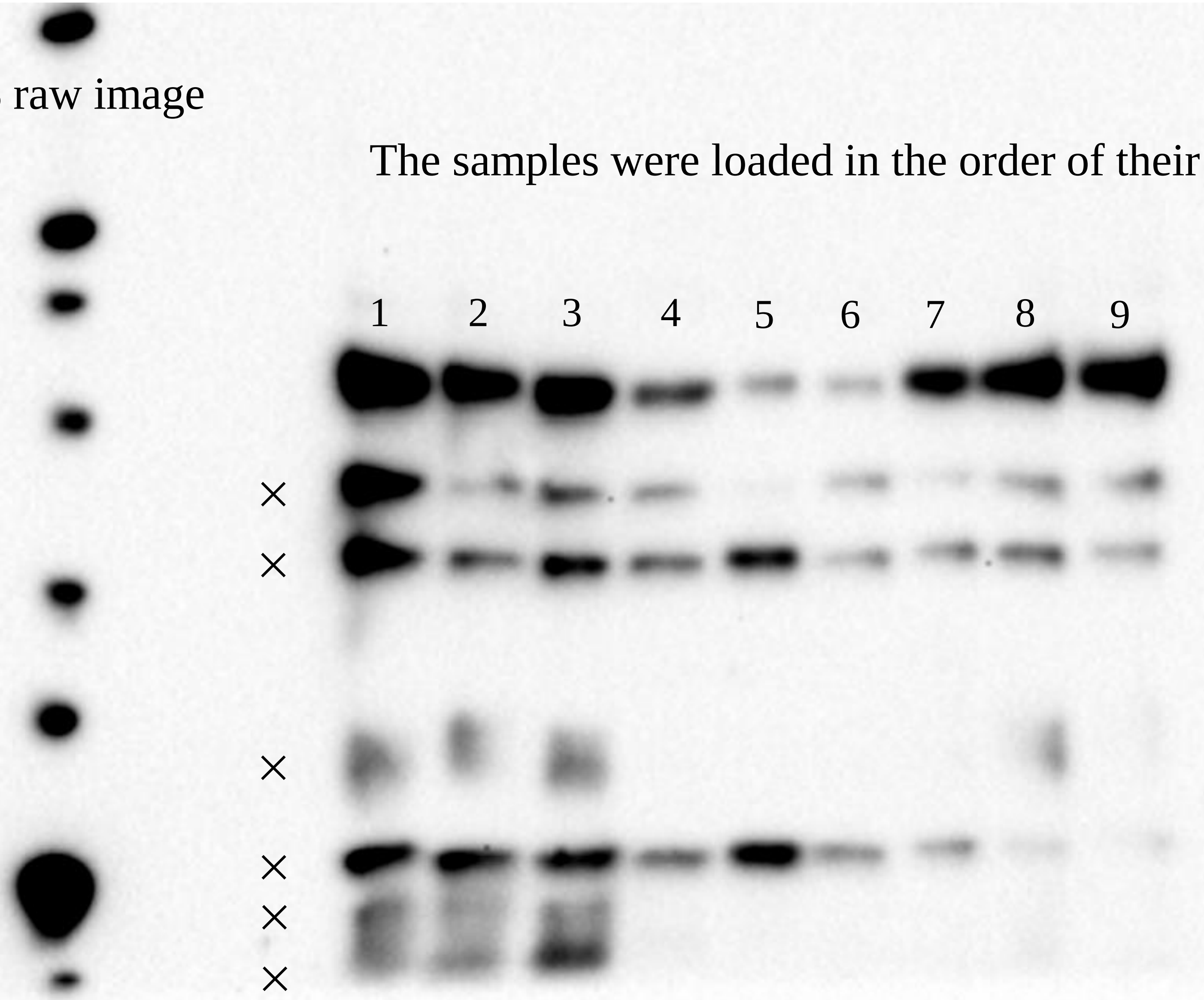

Antigen-antibody complexes were stained with Clarity Western ECL Substrate (Bio-Rad) and visualization was performed using an image scanner (ChemiDoc XRS Plus Imaging System, Bio-Rad).

Sample ID	
1	2015051901
2	2015051902
3	2015051903
4	2018022807
5	2018022808
6	2018022809
7	2018022810
8	2018022811
9	2018022812

Fig8 raw image

The samples were loaded in the order of their numbers.

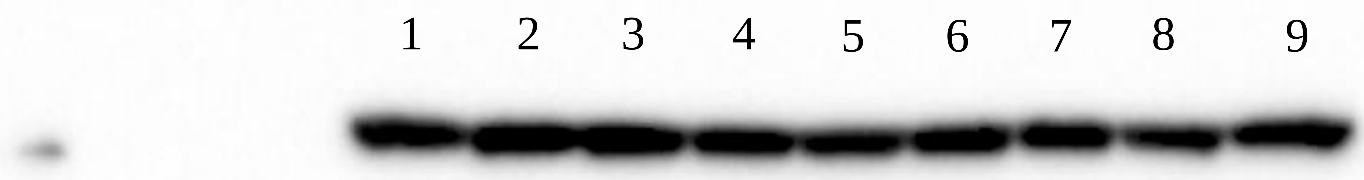

Antigen-antibody complexes were stained with Clarity Western ECL Substrate (Bio-Rad) and visualization was performed using an image scanner (ChemiDoc XRS Plus Imaging System, Bio-Rad).

Sample ID	
1	2015051901
2	2015051902
3	2015051903
4	2018022807
5	2018022808
6	2018022809
7	2018022810
8	2018022811
9	2018022812

S2 Fig raw image

The samples were loaded in the order of their numbers.

Sample ID	
1	2017120501
2	2017120502
3	2017120503
4	2015051910
5	2017120505
6	2017120505

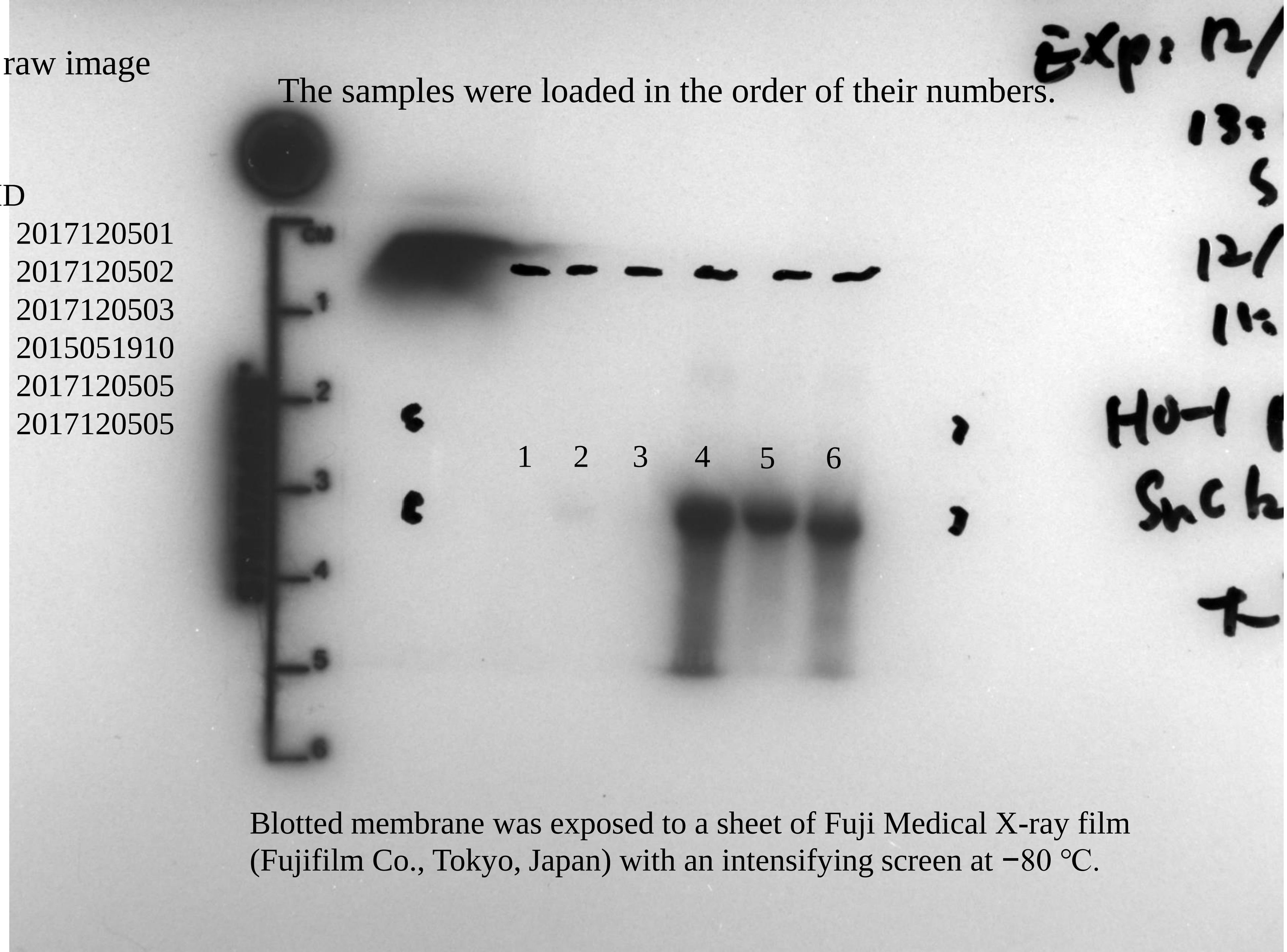

Blotted membrane was exposed to a sheet of Fuji Medical X-ray film (Fujifilm Co., Tokyo, Japan) with an intensifying screen at −80 ℃.

S2 Fig raw image

The samples were loaded in the order of their numbers.

Sample ID

1 2017120501
2 2017120502
3 2017120503
4 2015051910
5 2017120505
6 2017120505

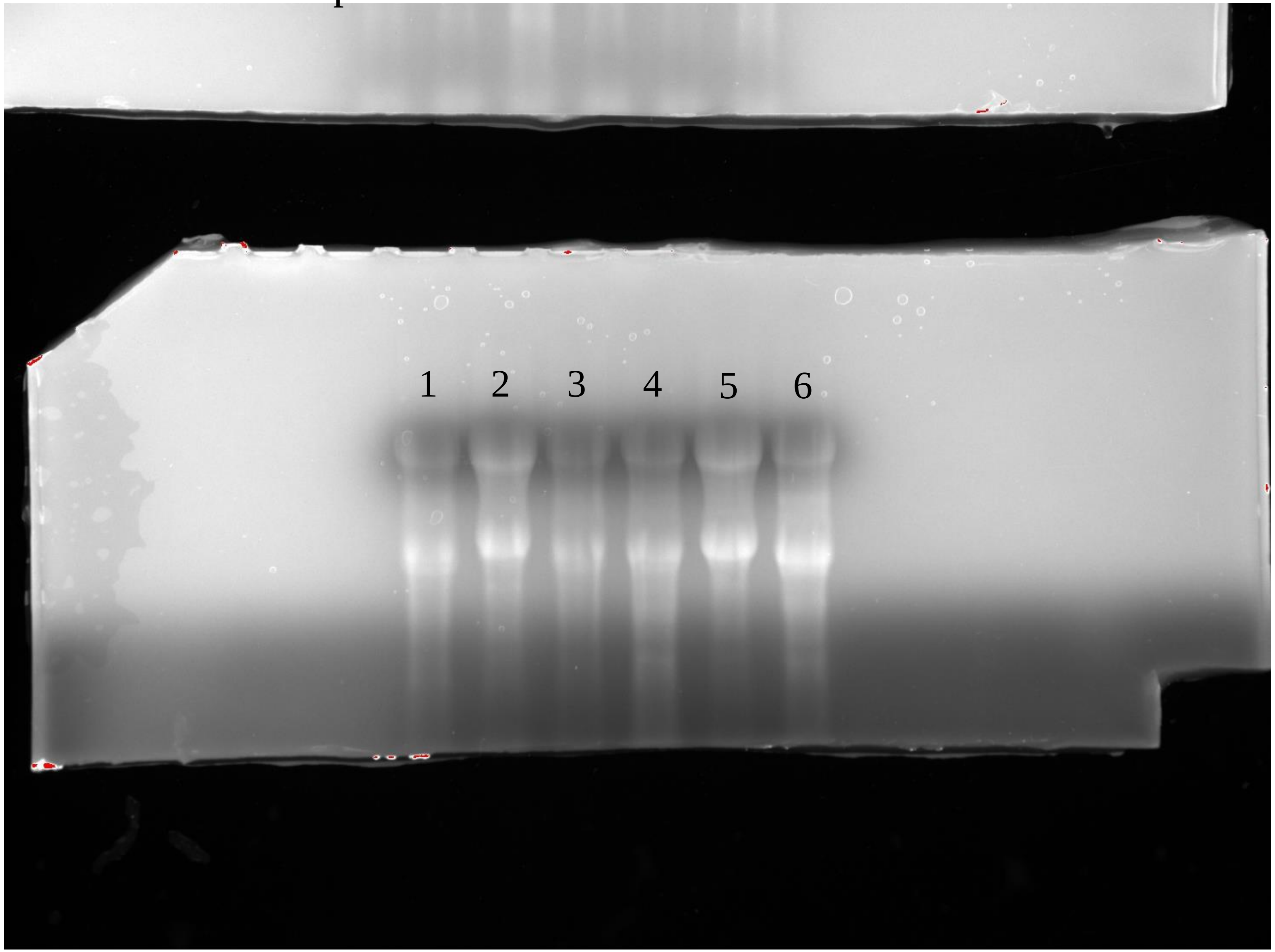

Signals corresponding to the target mRNA on the film and 18S ribosomal RNA bands visualized on the gel were estimated using an image scanner (ChemiDoc XRS Plus Image Processing System, Bio-Rad, USA) and image analysis software (Image Lab™ version 5.0; Bio-Rad Laboratories).

S3 Fig raw image

The samples were loaded in the order of their numbers.

Antigen-antibody complexes were stained with Clarity Western ECL Substrate (Bio-Rad) and visualization was performed using an image scanner (ChemiDoc XRS Plus Imaging System, Bio-Rad).

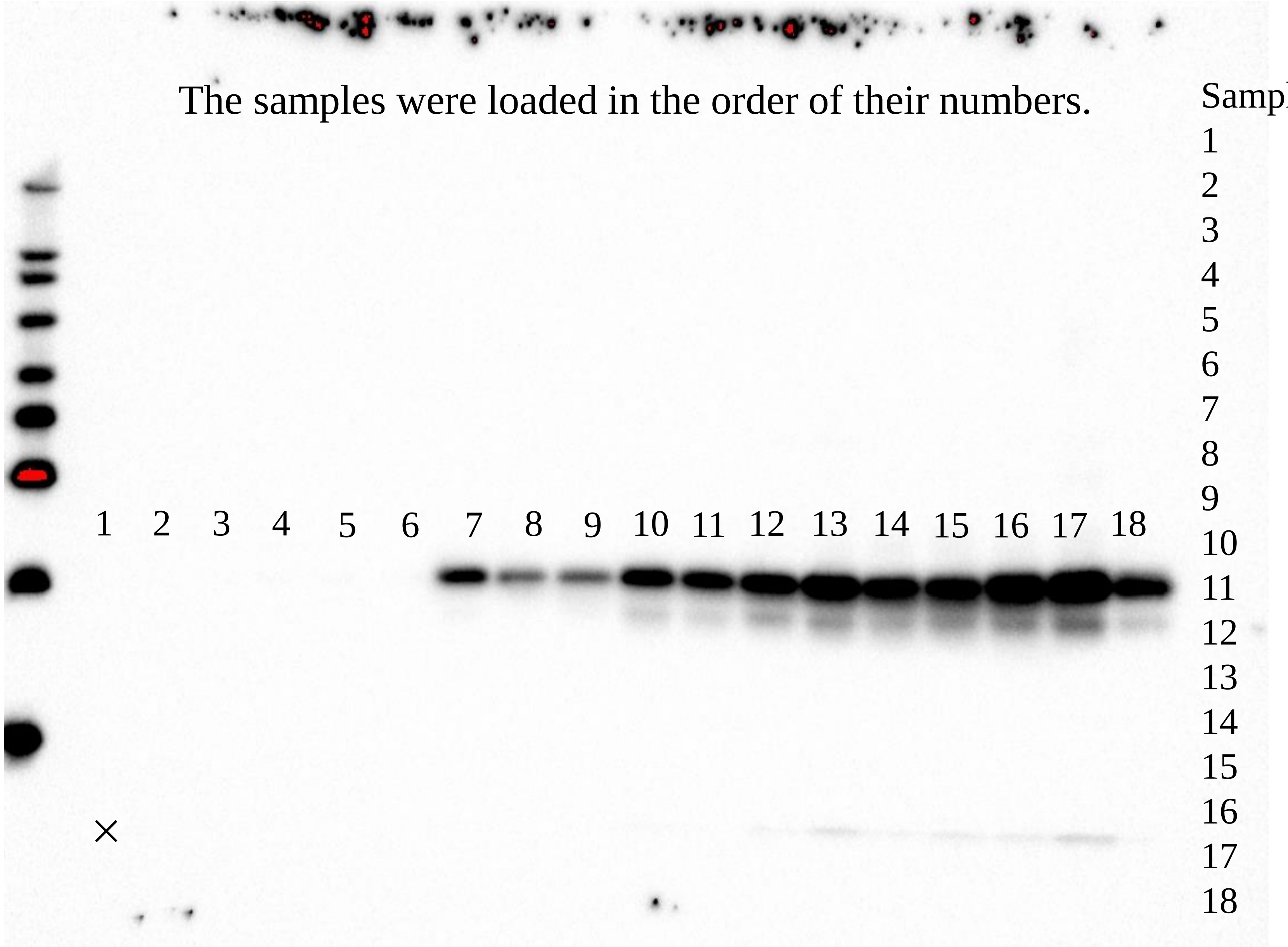

Sample ID	
1	2015051903
2	2015051902
3	2018022012
4	2017112206
5	2017112207
6	2017112208
7	2017112202
8	2017112203
9	2017112204
10	2017120704
11	2017120705
12	2018070502
13	2017120504
14	2017120505
15	2017120506
16	2018022804
17	2018022805
18	2018022806

S3 Fig raw image

Antigen-antibody complexes were stained with Clarity Western ECL Substrate (Bio-Rad) and visualization was performed using an image scanner (ChemiDoc XRS Plus Imaging System, Bio-Rad).

The samples were loaded in the order of their numbers.

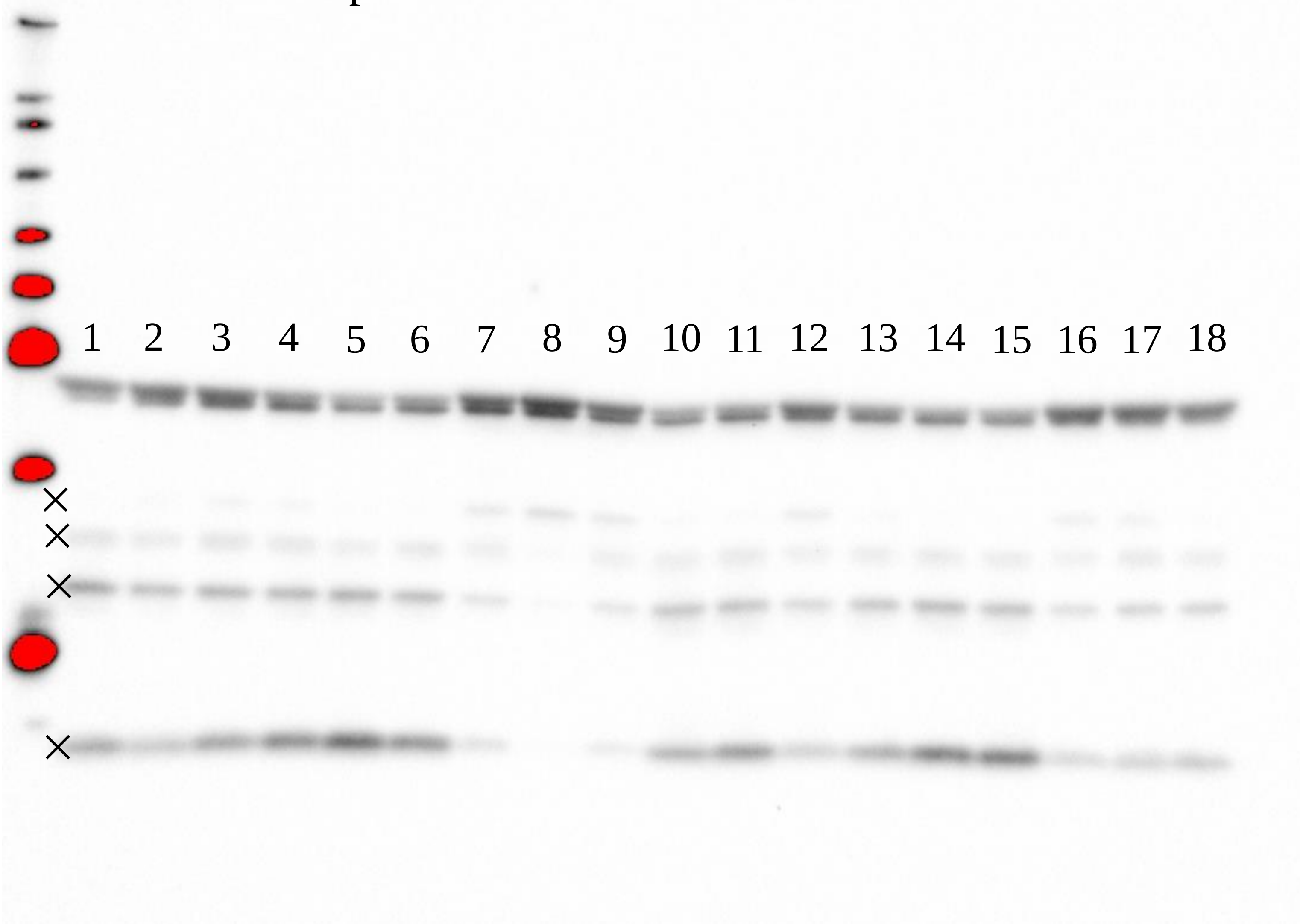

Sample ID	
1	2015051903
2	2015051902
3	2018022012
4	2017112206
5	2017112207
6	2017112208
7	2017112202
8	2017112203
9	2017112204
10	2017120704
11	2017120705
12	2018070502
13	2017120504
14	2017120505
15	2017120506
16	2018022804
17	2018022805
18	2018022806

S4 Fig raw image

The samples were loaded in the order of their numbers.

Sample ID	
1	2017112207
2	2017112203
3	2017120705
4	2017120506
5	2018022805

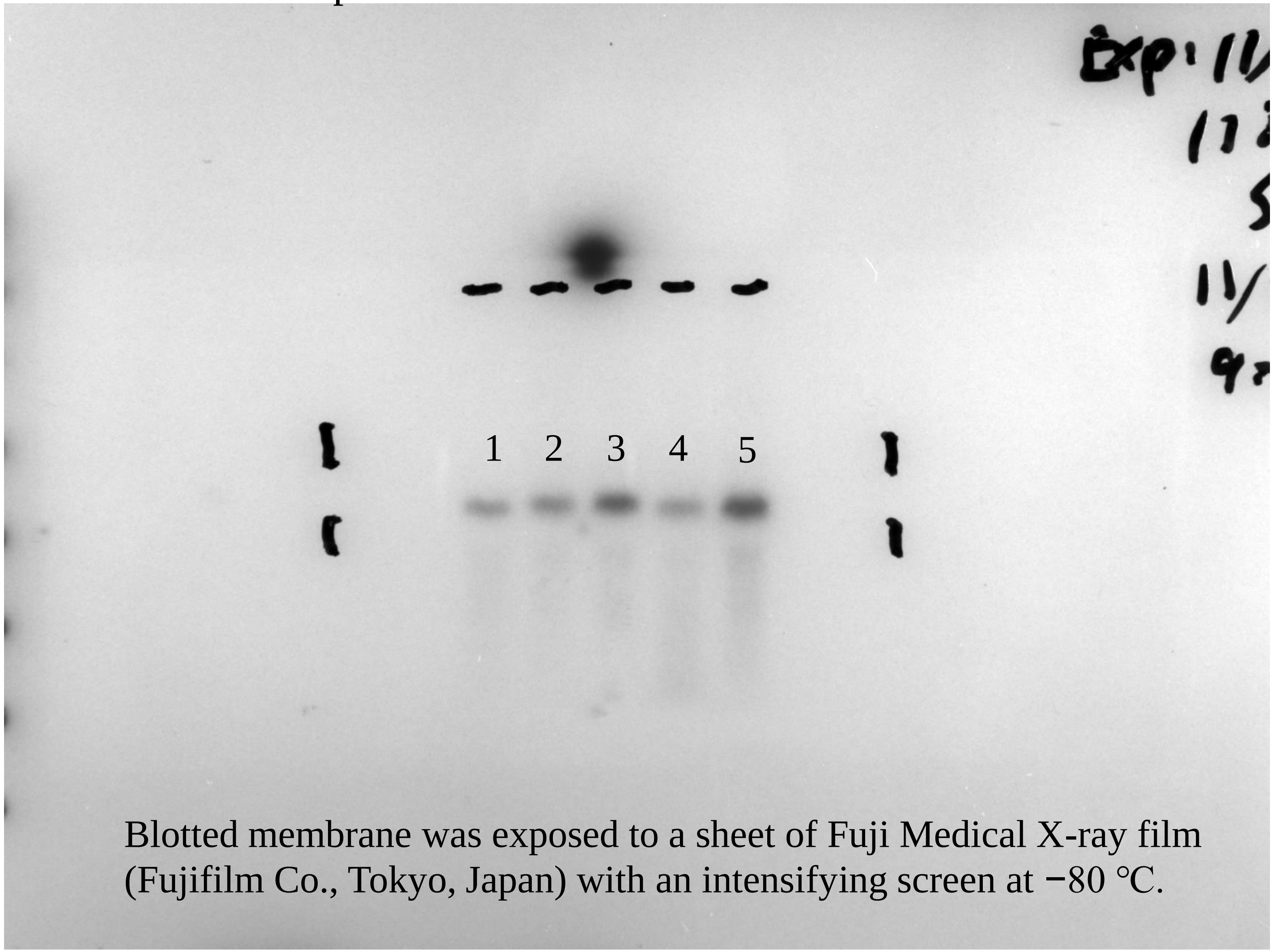

Blotted membrane was exposed to a sheet of Fuji Medical X-ray film (Fujifilm Co., Tokyo, Japan) with an intensifying screen at −80 °C.

S4 Fig raw image

The samples were loaded in the order of their numbers.

Sample ID	
1	2017112207
2	2017112203
3	2017120705
4	2017120506
5	2018022805

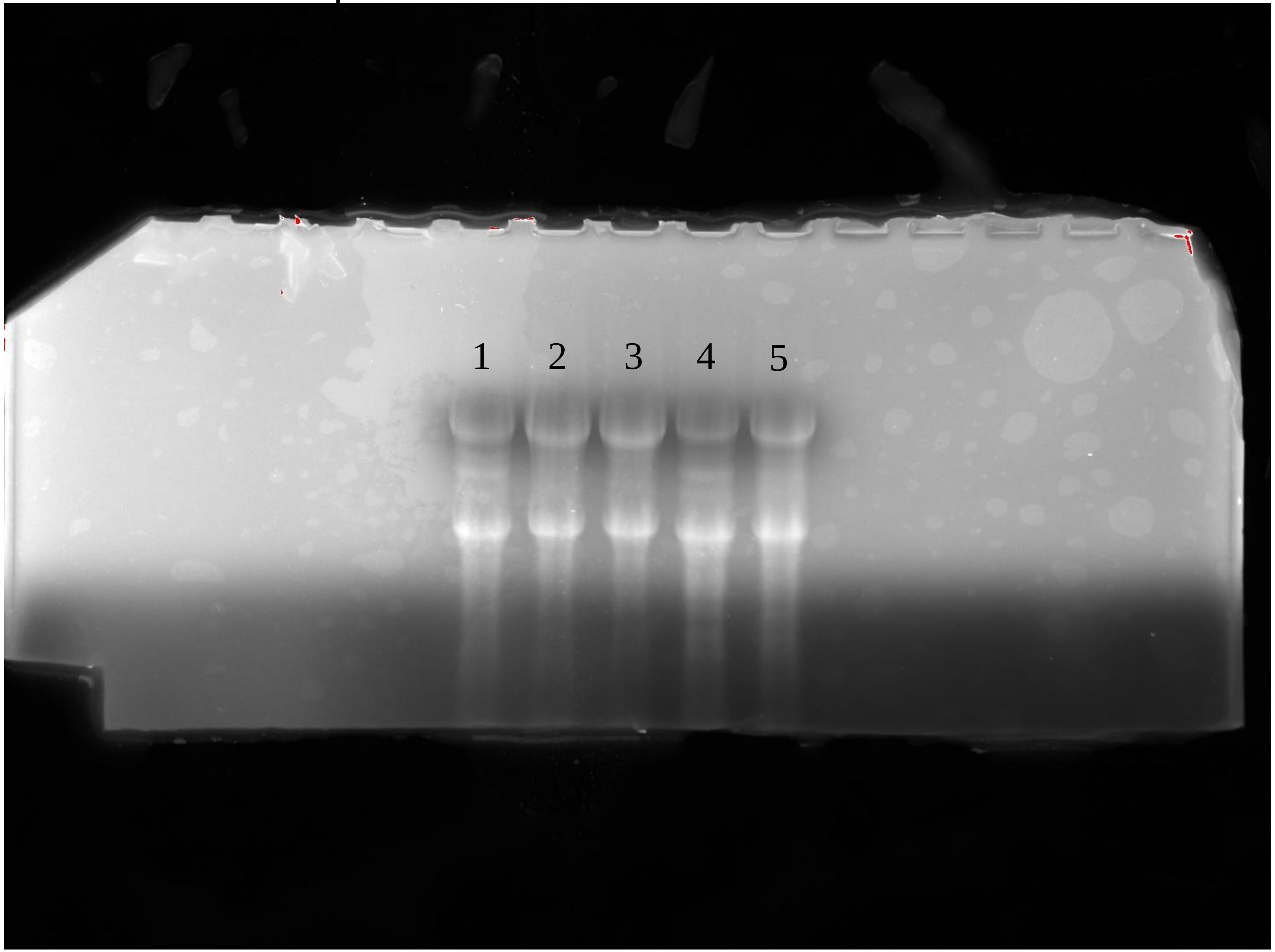

Signals corresponding to the target mRNA on the film and 18S ribosomal RNA bands visualized on the gel were estimated using an image scanner (ChemiDoc XRS Plus Image Processing System, Bio-Rad, USA) and image analysis software (Image Lab™ version 5.0; Bio-Rad Laboratories).

S5 Fig raw image

Antigen-antibody complexes were stained with Clarity Western ECL Substrate (Bio-Rad) and visualization was performed using an image scanner (ChemiDoc XRS Plus Imaging System, Bio-Rad).

The samples were loaded in the order of their numbers.

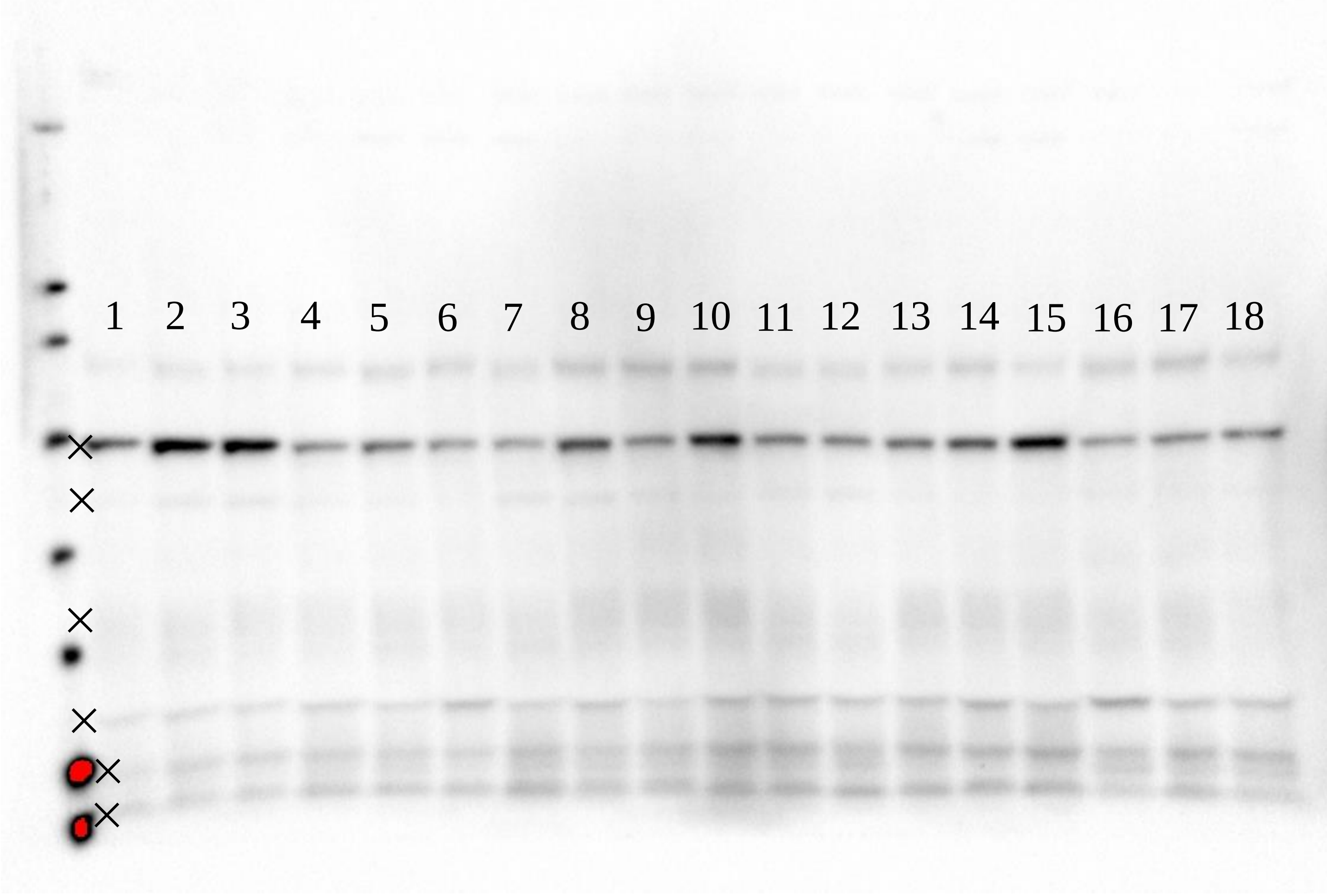

Sample ID	
1	2015051903
2	2015051902
3	2018022012
4	2017112206
5	2017112207
6	2017112208
7	2017112202
8	2017112203
9	2017112204
10	2017120704
11	2017120705
12	2018070502
13	2017120504
14	2017120505
15	2017120506
16	2018022804
17	2018022805
18	2018022806

S5 Fig raw image

Antigen-antibody complexes were stained with Clarity Western ECL Substrate (Bio-Rad) and visualization was performed using an image scanner (ChemiDoc XRS Plus Imaging System, Bio-Rad).

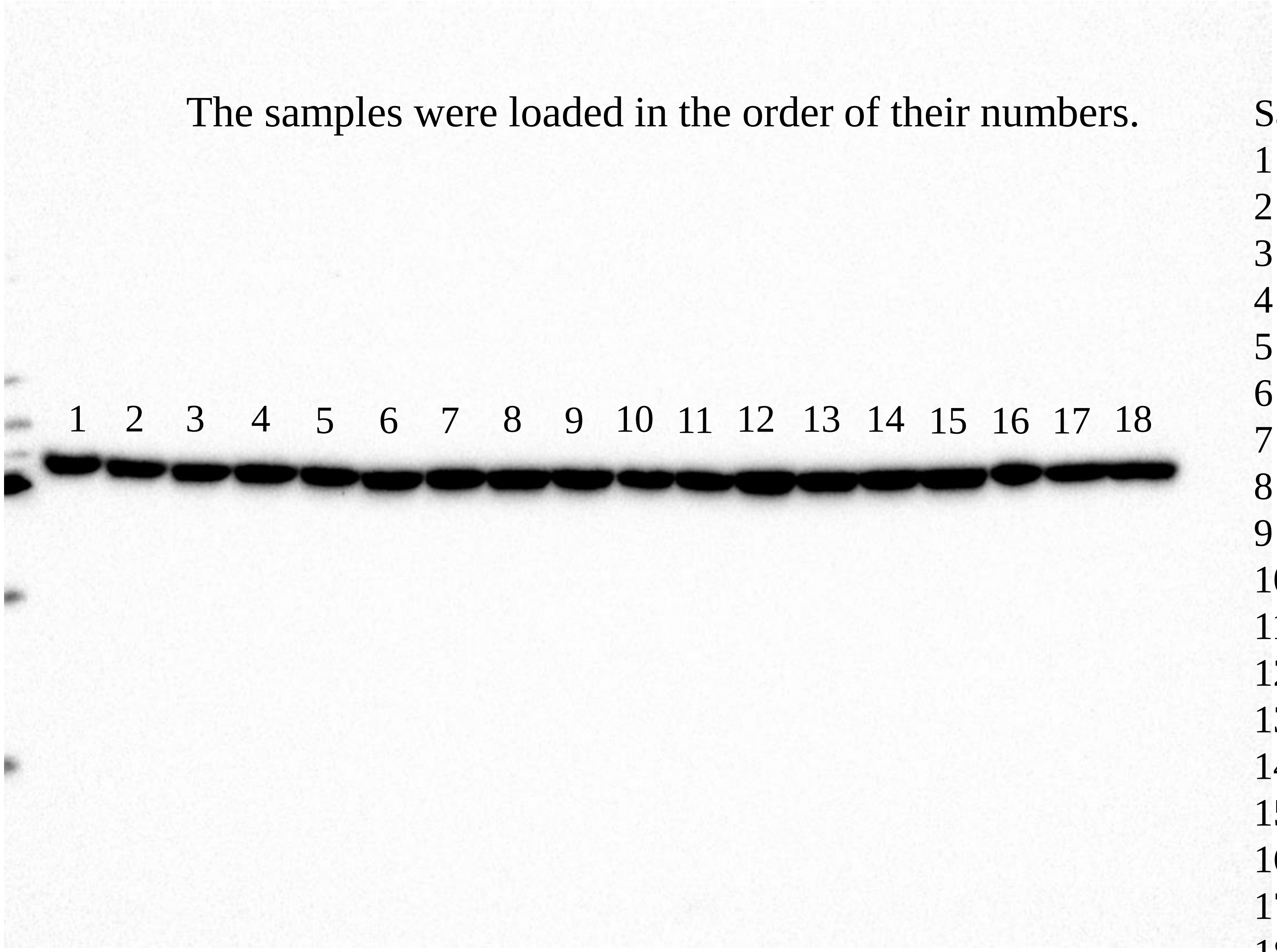

Sample ID	
1	2015051903
2	2015051902
3	2018022012
4	2017112206
5	2017112207
6	2017112208
7	2017112202
8	2017112203
9	2017112204
10	2017120704
11	2017120705
12	2018070502
13	2017120504
14	2017120505
15	2017120506
16	2018022804
17	2018022805
18	2018022806
